# Concomitant COX-1 and COX-2 suppression is not sufficient to induce enteropathy associated with chronic NSAID use

**DOI:** 10.1172/JCI190575

**Published:** 2026-01-27

**Authors:** Kayla Barekat, Soumita Ghosh, Christin Herrmann, Karl Keat, Charles-Antoine Assenmacher, Ceylan Tanes, Naomi Wilson, Ronan Lordan, Antonijo Mrčela, Lubica Rauova, Arjun Sengupta, Ujjalkumar Subhash Das, Robin Joshi, Elliot Friedman, Marylyn D. Ritchie, Kyle Bittinger, Aalim Weljie, Ken Cadwell, Frederic D. Bushman, Gary D. Wu, Garret A. FitzGerald, Emanuela Ricciotti

**Affiliations:** 1 Institute for Translational Medicine and Therapeutics,; 2 Department of Medicine, Division of Gastroenterology and Hepatology, and; 3 Division of Informatics, Department of Biostatistics, Epidemiology, and Informatics, Perelman School of Medicine, University of Pennsylvania, Philadelphia, Pennsylvania, USA.; 4 Department of Pathobiology, School of Veterinary Medicine, University of Pennsylvania, Philadelphia, Pennsylvania, USA.; 5 Department of Pediatrics, Division of Gastroenterology, Hepatology, and Nutrition, and; 6 Department of Pediatrics, Division of Hematology, Children’s Hospital of Philadelphia, Philadelphia, Pennsylvania, USA.; 7 Department of Systems Pharmacology and Translational Therapeutics,; 8 Department of Microbiology, and; 9 Department of Medicine, Perelman School of Medicine, University of Pennsylvania, Philadelphia, Pennsylvania, USA.

**Keywords:** Gastroenterology, Inflammation, Therapeutics, Eicosanoids

## Abstract

NSAIDs are the most widely used medications for the management of chronic pain; however, they are associated with numerous gastrointestinal (GI) adverse events. Although many mechanisms have been suggested, NSAID-induced enteropathy is thought to be primarily due to inhibition of both COX-1 and -2, which results in suppression of prostaglandin synthesis. Yet surprisingly, we found that concomitant postnatal deletion of *Cox-1* and *-2* over 10 months failed to cause intestinal injury in mice unless they were treated with naproxen or its structural analog, phenylpropionic acid, which is not a COX inhibitor. *Cox* double-knockout mice exhibited a distinct gut microbiome composition, and cohousing them with controls rescued their dysbiosis and delayed the onset of NSAID-induced GI bleeding. In both the UK Biobank and All of Us human cohorts, coadministration of antibiotics with NSAIDs was associated with an increased frequency of GI bleeding. These results showed that prostaglandin suppression played a trivial role in NSAID-induced enteropathy. However, *Cox* deletion caused dysbiosis of the gut microbiome, which amplified the enteropathic response to NSAIDs.

## Abstract 2

Simultaneous deletion of cyclooxygenases-1 and -2 does not cause spontaneous intestinal injury but increases the susceptibility of mice to severe lesions when treated with NSAIDs.

## Introduction

NSAIDs are the most widely used medications for the management of chronic pain and inflammation, with tens of millions of people worldwide consuming them daily (1, 2). NSAIDs are not addictive like opiates, but they are still associated with gastrointestinal (GI) (3) and cardiovascular (4) adverse events. Endoscopic studies have estimated the prevalence of inflammation in 60%–70%, blood loss and anemia in 30%, and mucosal ulceration in 30%–40% of chronic users (5, 6). Notably, there is a wide range of interindividual variation in response to NSAIDs – in both safety and efficacy – which cannot be fully explained by genetic differences in host metabolizing enzymes (7, 8). While the mechanisms underlying NSAID-induced gastropathy are well established (9–17). NSAID-induced enteropathy remains poorly understood, and there is still no treatment or preventative solution for the intestinal symptoms.

The antiinflammatory, analgesic efficacy of NSAIDs is attributable to suppression of prostaglandins by COX-1 and -2 (18, 19). COX-1 is the more constitutively expressed isoform, while COX-2 is induced by inflammatory and mitogenic stimuli (20). However, these distinctions are not absolute: COX-1 can be upregulated under certain circumstances (21–24), and COX-2 is constitutively expressed in some tissues (25–29). Enteropathy has been attributed to inhibition of COX-1–dependent synthesis of prostaglandin (PG) E_2_ and prostacyclin (PGI_2_) in intestinal epithelial cells, which are involved in protection of the mucosal barrier (30). This is compounded by inhibition of COX-1–dependent thromboxane (Tx) A_2_ formation in platelets, predisposing to hemorrhage (31). However, pharmacological inhibition or genetic deletion of COX-1 alone does not result in spontaneous enteric lesions (32, 33). Conventional deletion of both COXs is embryonically lethal, but combined genetic deletion of one and pharmacological inhibition of the other (as well as pharmacological inhibition of both) result in enterotoxicity (33–35). COX-2–derived PGs also play key roles in intestinal mucosal defense (36) and injury repair (37, 38). Yet similar to the case of COX-1 suppression alone, COX-2 suppression alone results in little to no enteric injury relative to nonselective NSAIDs in mice (39–42). In humans, clinical trials in arthritis have shown that selective inhibition of COX-2 roughly halves the incidence of GI bleeds compared with mixed inhibitors (43, 44), and although expression of both enzymes is upregulated in inflammatory synovia (45), comparative efficacy trials of selective versus nonselective NSAIDs have not been performed.

While PG suppression has been thought to be the dominant cause of NSAID enteropathy, additional factors, including direct chemical toxicity (46–48), disruption of mitochondrial function (49, 50), intestinal dysbiosis (51–57), and bile acid toxicity (58–60), have also been implicated. To address further the mechanisms that underlie NSAID enteropathy, we developed 2 reagents: (a) mice in which deletion of both enzymes is achieved postnatally, thereby circumventing the critical role of COXs in pregnancy and embryonic development (61, 62); and (b) a chronic dosing model with the NSAID naproxen, where drug exposure and PG suppression correspond to what is seen in humans (63, 64). To our surprise, there was minimal evidence of enteropathy consequent to *Cox* deletion, whereas naproxen-induced GI toxicity was amplified in the double knockouts (DKOs). As PG suppression was comparable between the DKOs and naproxen treatment, it is not the explanation for the NSAID enterotoxicity. Rather, drug interactions with the gut microbiome are of more importance.

## Results

### Inducible Cox-1 and -2 deletion mouse model demonstrates that chronic PG depletion alone does not result in spontaneous GI injury.

**Figure 1:**
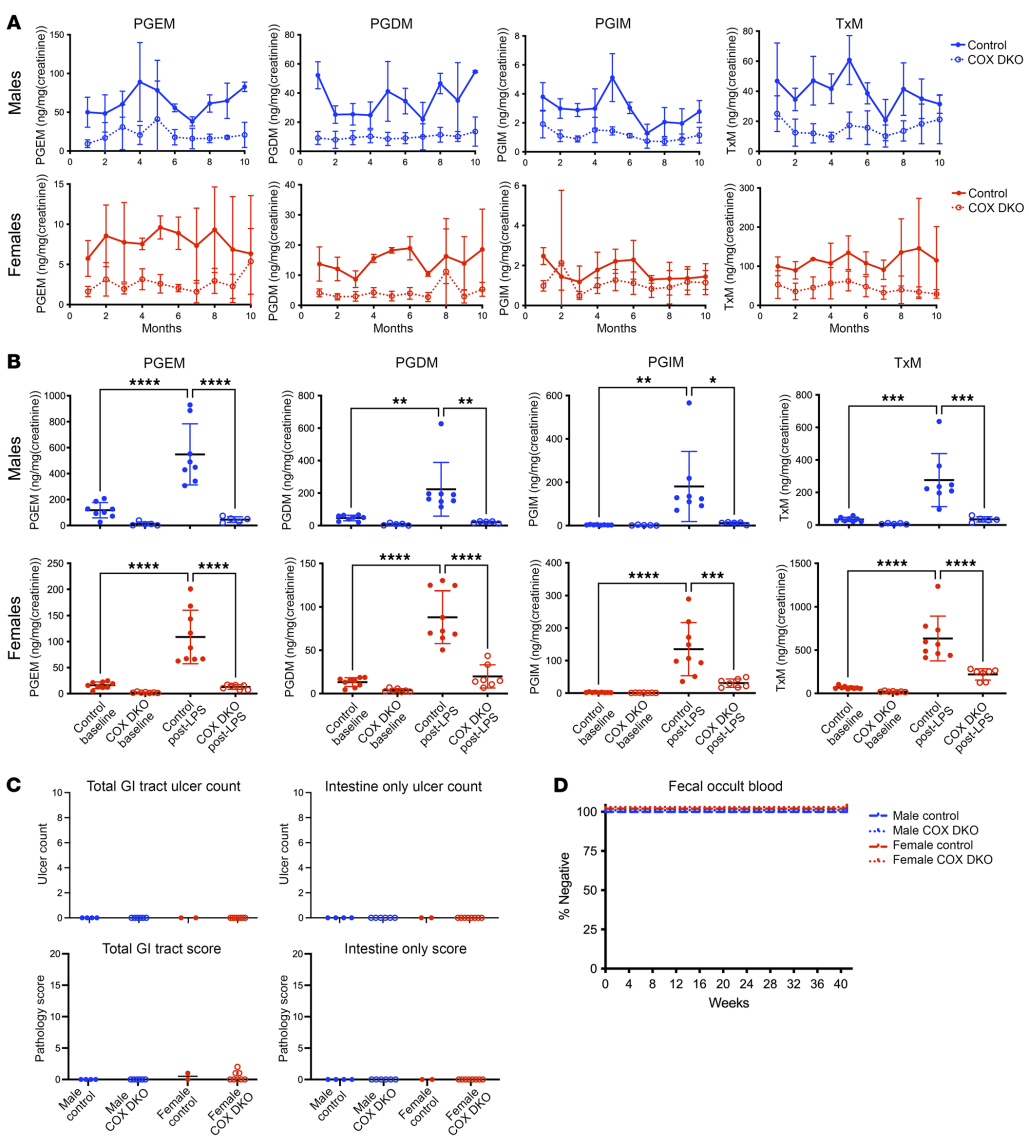
Postnatal deletion of *Cox-1* and *-2* reveals that chronic PG depletion alone is not sufficient to induce spontaneous GI injury in mice. (**A**) Longitudinal tracking of universal inducible *Cox*-DKO mice was conducted over 10 months after tamoxifen exposure (administered at 10–12 weeks of age). Monthly urinary PGI_2_ (PGIM), PGD_2_ (PGDM), PGE_2_ (PGDM), and thromboxane (TxM) metabolite levels measured by LC-MS/MS, analyzed separately for each sex. *n* = 14 *Cox*-DKO mice and 6 *Cre^–/–^* controls. (**B**) Attempted induction of PG synthesis in *Cox*-DKO mice via LPS challenge. Mice received 1 mg/kg BW LPS by i.p. injection, and urine was collected overnight. *n* = 7–9 mice per group. Urinary PG metabolites measured by LC-MS/MS for male and female *Cox*-DKO mice and *Cre^–/–^* controls at baseline versus post-LPS exposure. *P < 0.05, **P < 0.01, ***P < 0.001, ****P < 0.0001 by 1-way ANOVA. (**C**) Total ulcer count throughout the entire GI tract (including stomach, small intestine, and large intestine), as counted by a blinded pathologist, paired with qualitative/semiquantitative pathology scores throughout the entire GI tract for longitudinal tracking experiment. Each data point represents a single mouse. (**D**) Weekly hemoccult test results plotted as a Kaplan-Meier curve for percentage of each group that tested negative for blood in the stool. Any individual that tested positive was marked positive for that first week and all subsequent weeks, resembling a survival curve.

Upon confirmation that *Cox-1* and *-2* are expressed in human epithelial cells (Supplemental Figure 1, A and B; supplemental material available online with this article; https://doi.org/10.1172/JCI190575DS1), we developed a tamoxifen-inducible universal Cre-recombinase mouse line with *loxP* sites flanking both *Cox-1* and *-2*. We first confirmed that these *Cox-1^fl/fl^ Cox-2^fl/fl^ CMV-Cre^+/–^* (*Cox*-DKO) mice exhibited reduced expression of the genes in 2 separate tissues (Supplemental Figure 2A), as well as reduced protein levels in small intestine under stimulated conditions (Supplemental Figure 2, B–E). We next confirmed PG suppression in vivo in the DKOs by measuring urinary metabolites over a sustained period (Figure 1A). This difference from *Cre^–/–^* control littermates was evoked further by stimulation of PG synthesis with LPS, which induces *Cox* expression (Figure 1B). Blinded assessment of tissue histology (Table 1 and Supplemental Figure 3) failed to detect GI ulcers or visible signs of enteropathy (Figure 1C) 10 months after tamoxifen-induced genetic deletion (~1 year of age) in the DKOs. Furthermore, no blood was detected in the stool of any individual mouse with weekly hemoccult testing (Figure 1D). Thus, marked suppression of PG synthesis alone does not result in gastroenteropathy.

### Chronic naproxen treatment in WT mice recapitulates signs of NSAID-induced enteropathy observed in human patients.

Naproxen is a nonselective NSAID that can elicit GI injury in both mice and humans, while celecoxib, selective for inhibition of COX-2, is associated with a reduced GI toxicity profile (44). Naproxen was chosen for our chronic NSAID dosing model because it is commonly used for long-term daily treatment in patients with osteoarthritis and rheumatoid arthritis, owing to its long half-life and relatively favorable cardiovascular profile (63). To establish our chronic NSAID dosing model, C57BL/6 WT mice were fed a custom diet with either naproxen (230 mg/kg), celecoxib (100 mg/kg), or no drug (control) dry-mixed into it and were allowed to feed ad libitum for 3 full weeks prior to tissue collection. Plasma drug concentrations at steady state for both NSAIDs corresponded to human systemic drug exposure under chronic dosing conditions (63–66) (Supplemental Figure 4A). As reported in humans, mice treated with naproxen — but not those treated with celecoxib — showed a significant reduction in serum TxB_2_ (Supplemental Figure 5A). The half-life of naproxen in mice is approximately 7.5 hours (Supplemental Figure 5B).

**Figure 2:**
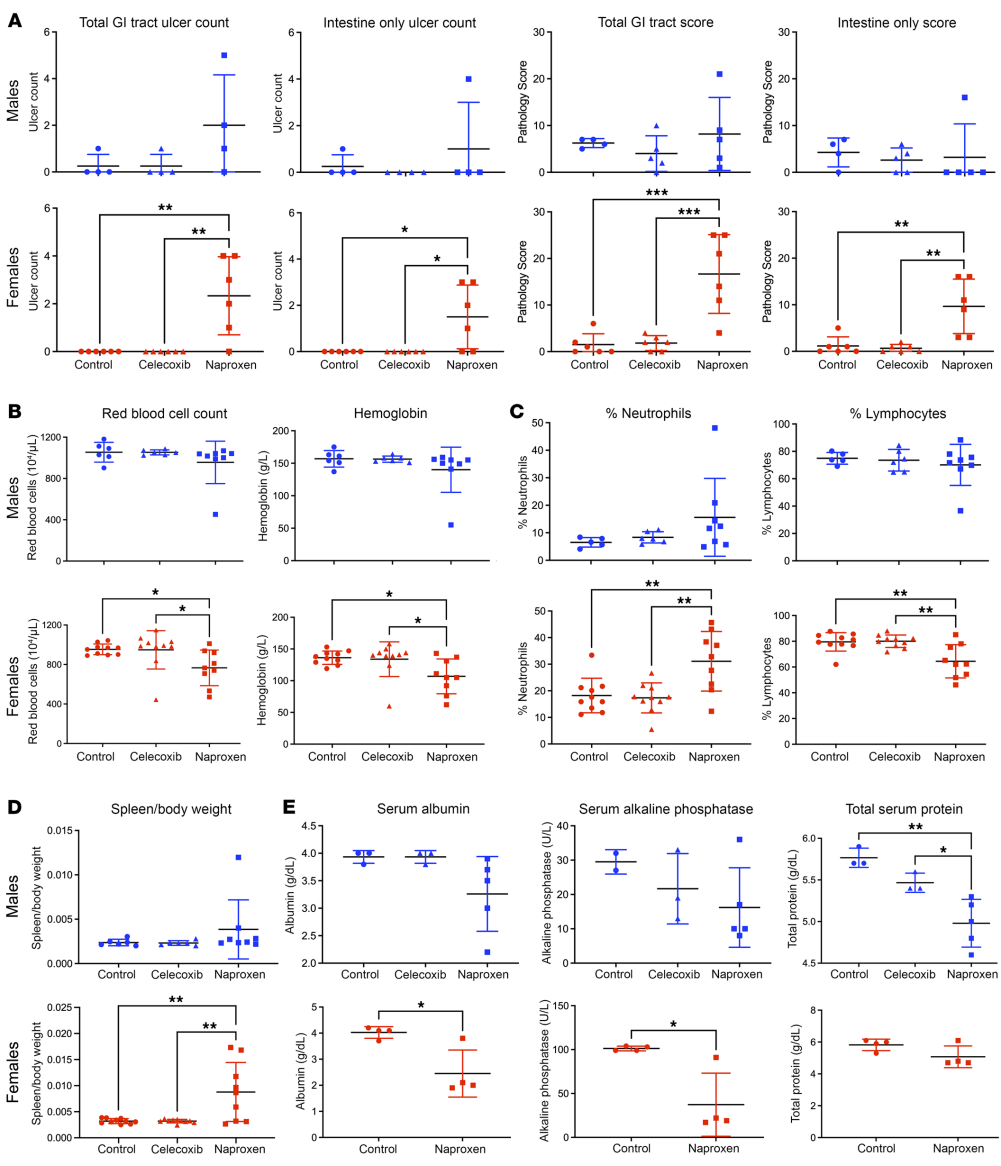
Chronic naproxen dosing in mice mimics NSAID enteropathy in humans. WT C57BL/6J mice were treated with either control diet, celecoxib diet (100 mg/kg), or naproxen diet (230 mg/kg) and allowed to feed ad libitum for 3 weeks prior to tissue collection. (**A**) Ulcer count for total GI tract versus small intestine only as well as pathology score for total GI tract versus small intestine only; males and females are graphed separately. *n* = 4–6 mice per group. *P < 0.05, **P < 0.01, ***P < 0.001 by 1-way ANOVA. (**B**) Red blood cell count and hemoglobin measured by complete blood count (CBC); males and females are graphed separately. *n* = 6–9 mice per group. *P < 0.05 by 1-way ANOVA. (**C**) Percentage of neutrophils and lymphocytes relative to all white blood cells measured by CBC; males and females are graphed separately. *n* = 6–9 mice per group. **P < 0.01 by 1-way ANOVA. (**D**) Spleen weight relative to total BW; males and females are graphed separately. *n* = 6–9 mice per group. **P < 0.01 by 1-way ANOVA. (**E**) Serum proteins and liver enzymes measured by clinical automated chemistry analyzer; males and females are graphed separately. *n* = 3–5 mice per group. *P < 0.05, **P < 0.01 by 1-way ANOVA (top) or unpaired 1-tailed *t* test (bottom).

Within 3 weeks of exposure, naproxen (but not celecoxib) caused GI ulcers and inflammation in some mice, more severely in females and mostly located in the small intestine (Figure 2A). Some of the mice with enteropathy showed signs of anemia (Figure 2B), neutrophilia and lymphopenia (Figure 2C), an increase in spleen weight relative to BW (Figure 2D), and decreases in albumin, alkaline phosphatase, and total serum proteins (Figure 2E). In summary, levels of systemic naproxen exposure that correspond to those attained clinically result in intestinal ulceration, intestinal and systemic inflammation, GI bleeding, anemia, and protein deficiency characteristic of NSAID-induced enteropathy in humans.

### Naproxen-induced enteropathy is more severe on Cox-DKO background.

**Figure 3:**
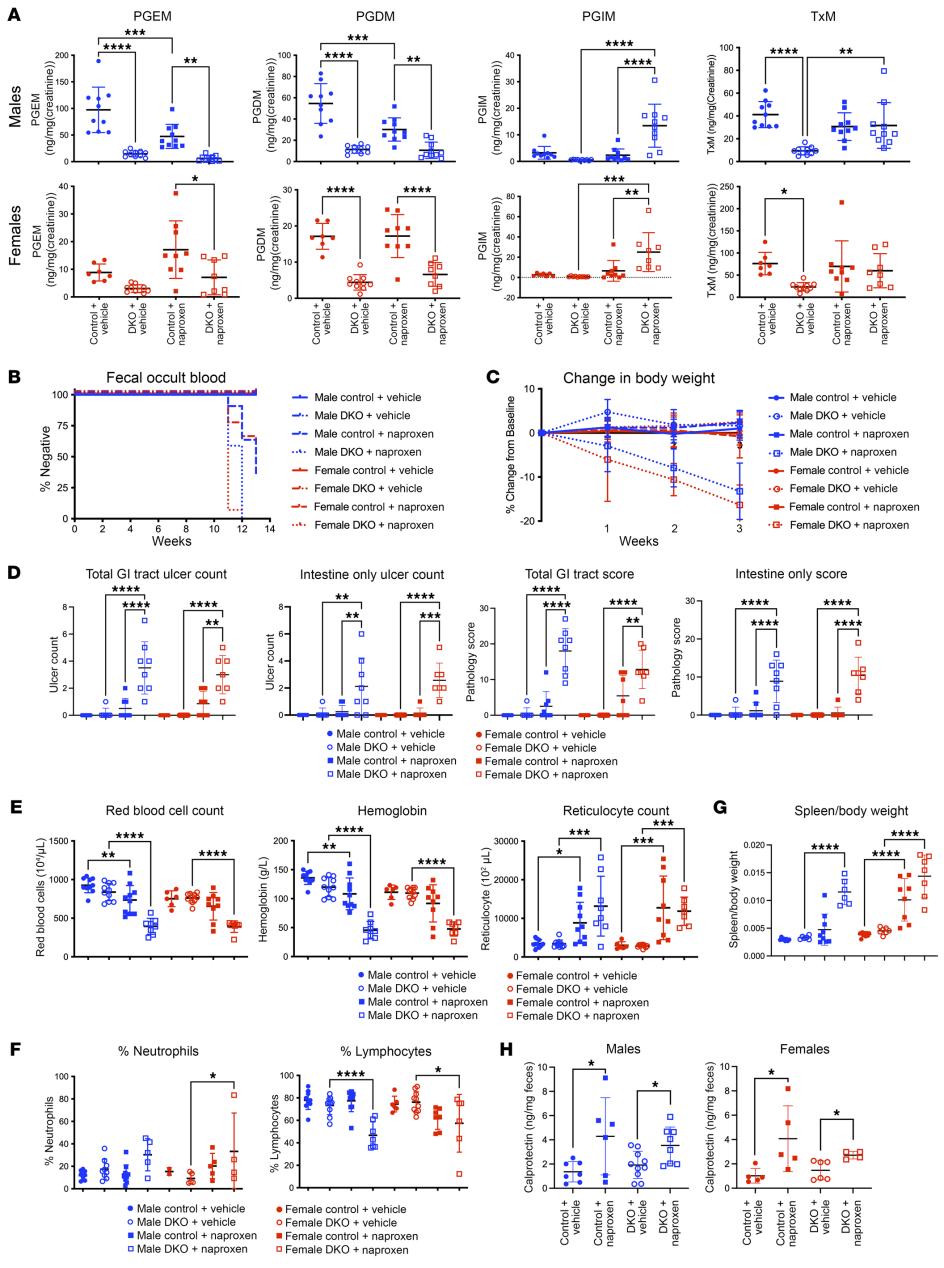
Naproxen enteropathy is exacerbated in *Cox*-DKO mice. *Cox*-DKO and *Cre^–/–^* control mice were treated with either control diet or naproxen diet (230 mg/kg) and allowed to feed ad libitum for 3 weeks prior to tissue collection. *n* = 7–10 mice per group for entire figure. (**A**) Urinary PG metabolites measured by LC-MS/MS, analyzed separately for each sex. *P < 0.05, **P < 0.01, ***P < 0.001, ****P < 0.0001 by 1-way ANOVA. (**B**) Weekly hemoccult test results plotted as a Kaplan-Meier curve for percentage of each group that tested negative for blood in the stool. Any individual that tested positive was marked positive for that first week and all subsequent weeks, resembling a survival curve. (**C**) Percent change in BW relative to baseline BW. (**D**) Ulcer count for total GI tract versus small intestine only as well as pathology score for total GI tract versus small intestine only; males and females are graphed separately. **P < 0.01, ***P < 0.001, ****P < 0.0001 by 1-way ANOVA. (**E**) Red blood cells, hemoglobin, and reticulocytes measured by CBC. *P < 0.05, **P < 0.01, ***P < 0.001, ****P < 0.0001 by 1-way ANOVA. (**F**) Percentage of neutrophils and lymphocytes relative to all white blood cells measured by CBC. *P < 0.05, ****P < 0.0001 by 1-way ANOVA. (**G**) Spleen weight relative to total BW. ****P < 0.0001 by 1-way ANOVA. (**H**) Fecal calprotectin measured by ELISA. *P < 0.05 by 1-way ANOVA.

We next administered the same naproxen diet to *Cox*-DKO mice and *Cre^–/–^* control littermates. Urinary PGE_2_ and PGD_2_ metabolites were suppressed in both sexes to a similar degree by *Cox* deletion and naproxen treatment, while urinary Tx-M was depressed in both sexes in the DKOs but not on naproxen. Addition of naproxen to the DKO mice did not significantly further depress PG synthesis (Figure 3A). DKO mice exhibited blood in the stool (Figure 3B), weight loss (Figure 3C), and ulcers (Figure 3D) only when they were treated with naproxen. However, naproxen-induced GI injury was amplified when given on a background of *Cox* depletion. This was also true of indirect indices of GI blood loss: reticulocytosis and anemia (Figure 3E), neutrophilia and lymphopenia (Figure 3F), and increased spleen weight (Figure 3G). As an additional marker of GI inflammation, fecal calprotectin — a calcium-binding protein found in granulocytes that correlates closely with neutrophil infiltration of the intestinal mucosa — was found to be elevated only in mice treated with naproxen (Figure 3H).

As a further control, we repeated the chronic naproxen dosing experiment in DKO mice and *Cre^–/–^* littermates using (*R*)-(–)-2-phenylpropionic acid (PPA), which is a weakly acidic structural analog of naproxen that lacks its COX inhibitory property (67) and should thus be roughly equivalent to untreated *Cre^–/–^* mice in terms of urinary PG metabolite measurements. The same concentration of PPA (230 mg/kg) as naproxen was dry-mixed into the diet, and mice were allowed to feed ad libitum for 3 weeks prior to tissue harvest. As expected, PPA did not depress PG synthesis (Supplemental Figure 6A), but it did cause anemia (Supplemental Figure 6B) and enteropathy (Supplemental Figure 6C), again exacerbated by *Cox* depletion as observed with naproxen. Unlike naproxen, PPA did not cause weight loss (Supplemental Figure 6D). Many of the *Cox-*DKOs on PPA exhibited some degree of GI injury, whereas the *Cre^–/–^* controls were mostly unaffected. Of note, the injury induced by PPA was more heavily localized to the stomach than the small intestine. PPA does not undergo notable enterohepatic recirculation; therefore, it causes intestinal damage primarily through direct contact with the intestinal mucosa, but to a lesser extent than naproxen, which, in addition to directly damaging the intestinal mucosa, undergoes enterohepatic recirculation and inhibits protective PGs. In summary, although PG suppression alone – by *Cox* depletion – does not cause enteropathy, it enhances the sensitivity of the mice to enteropathy induced by naproxen or its structural analog PPA.

### Cox-DKO mice exhibit distinct gut microbiome composition at baseline, predisposing them to more severe enteropathy upon naproxen treatment.

**Figure 4:**
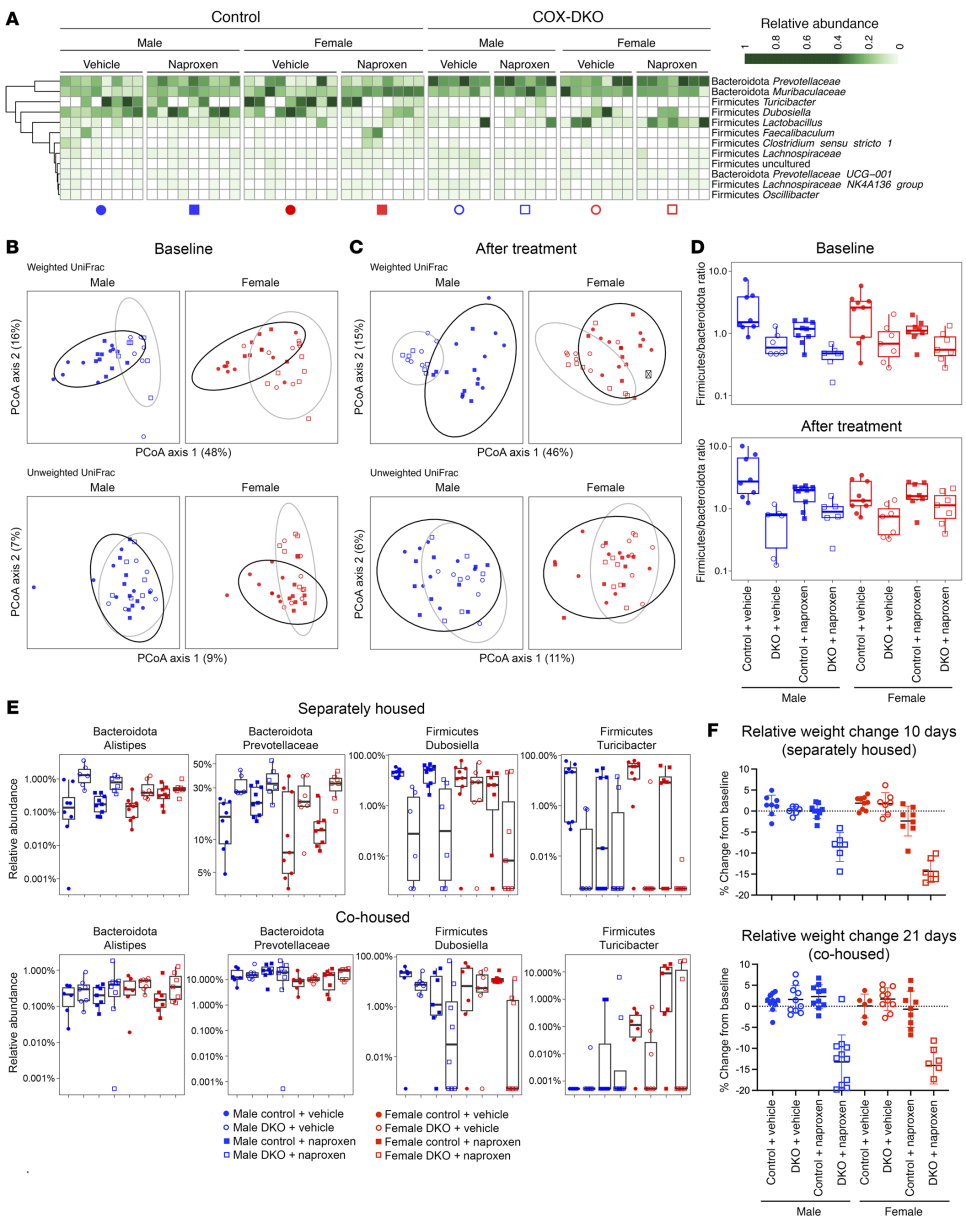
Divergent gut microbiome composition at baseline predisposes *Cox*-DKO mice to more severe enteropathy when treated with naproxen. *Cox*-DKO and *Cre^–/–^* control mice were treated with either control diet or naproxen diet (230 mg/kg) and allowed to feed ad libitum for either 10 days (separately housed by genotype) or 21 days (cohoused) prior to tissue collection; difference in duration was due to increased rate of naproxen-induced weight loss when mice were separately housed by genotype. *n* = 7–10 mice per group for entire figure. (**A**) Baseline bacterial taxa relative abundance heatmap including taxa at genus level or higher with an average relative abundance of at least 1% across all fecal samples. White tiles indicate no detection. (**B** and **C**) Beta diversity principal coordinate analysis PCoA plots of bacterial UniFrac distances at baseline (**B**) and after 10 days of naproxen treatment (**C**). Ellipses denote 95% confidence intervals on genotypes: control (black) and *Cox*-DKO (gray). (**D**) Box plots of the ratio of Firmicutes/Bacteroidota relative abundances at baseline (top) and after 10 days of naproxen treatment (bottom). (**E**) Box plots of differentially abundant taxa between genotypes at baseline. Top: separately housed animals. Bottom: cohoused animals. The box plots in **D** and **E** depict the minimum and maximum values (whiskers), the upper and lower quartiles, and the median. (**F**) Change in BW relative to baseline in separately housed (10 days) versus cohoused (21 days) experiments.

Given the extensive literature reports that pharmacological inhibition of COX isozymes is associated with dysbiosis in both rodents and humans (51, 53, 54, 57, 68–70), we performed 16S rRNA sequencing of feces from the DKOs and *Cre^–/–^* controls to investigate the effect of adult COX-1 and COX-2 deletion on the composition of the gut microbiome. Littermates were separated by genotype shortly after weaning, prior to tamoxifen exposure. The heatmap summary shows that the baseline gut microbiome differed by genotype (Figure 4A), with the *Cox*-DKOs displaying an increased relative abundance of *Prevotella* and a decreased relative abundance of *Turicibacter* and *Dubosiella*. Both sexes exhibited distinct microbiome composition by genotype when taking into account abundance of taxa (weighted UniFrac distance, male *P* = 0.001, female *P* = 0.005), but only females showed differences based on presence versus absence of taxa (unweighted UniFrac distance, male *P* = 0.08, female *P* = 0.02) (Figure 4B). After naproxen treatment, microbiome composition still differed by genotype for both the weighted and unweighted UniFrac distance metrics (Figure 4C; male *P* = 0.001, female *P* = 0.005 for weighted UniFrac; male *P* = 0.03, female *P* = 0.03 for unweighted UniFrac). Microbiome differences did not manifest with treatment alone, and the effect of genotype was independent of treatment. The Firmicutes/Bacteroidota ratio was also altered in the *Cox*-DKO mice (Figure 4D; baseline: male *P* = 0.004, female *P* = 0.03; after treatment: male *P* = 0.004, female *P* = 0.01).

The relative increase in Gram-negative relative to Gram-positive taxa in the *Cox*-DKO mice is consistent with previously published data on NSAID-induced enteropathy (51, 53, 54, 68–70). However, here, we associated this result with PG suppression in the absence of enteropathy and, unlike prior acute studies with other NSAIDs, we did not observe dysbiosis with chronic dosing of naproxen alone. High-abundance genera or families that were distinct between *Cox*-DKOs and control mice at baseline included *Turicibacter* (male *q* = 0.17, female *q* < 0.05), *Dubosiella* (male *q* < 0.05), *Prevotellaceae* (male *q* = 0.06, female *q* < 0.05), and *Alistipes* (male *q* < 0.05, female *q* = 0.1). However, these differential patterns were markedly dampened when littermate animals were not separated by genotype (all *q* > 0.05), shifting the *Cox-*DKO gut microbiome composition toward that of the controls (Figure 4E). No taxa met the FDR cutoff when littermates were not separated by genotype (Supplemental Figure 7, A and B). This cohousing of the *Cox*-DKOs with controls also delayed the onset of naproxen-induced weight loss (Figure 4F), suggesting a protective role of commensal microbiota (68, 70) from NSAID-associated enteropathy. These patterns remained consistent in species-level analyses. We found that *Lactobacillus murinus*, *Bacteroides uniformus*, *Bacteroides acidifaciens*, and an uncultured *Bacteroides* species were more abundant in the DKOs when separately housed by genotype (Supplemental Figure 8, A and B), but no statistically significant differences were detected at species level when cohoused (Supplemental Figure 8C).

### Chronic naproxen treatment elicits a distinct pattern in gut microbe–dependent bile acid conjugation, and suppression of bile acid synthesis is protective against naproxen-induced enteropathy.

We sought bacteria-derived metabolites of functional relevance that might result from the dysbiosis consequent to *Cox* depletion. A targeted measurement of entero-protective short-chain fatty acids (Supplemental Figure 9A) and an untargeted screen of metabolites (Supplemental Figure 9B) failed to identify candidates. Similarly, *Cox* depletion did not impact immune cells, as measured by flow cytometry, that could predispose the mice to exacerbate NSAID-induced enteropathy; proinflammatory patterns in myeloid cells that did not discriminate by genotype were only detected with naproxen challenge (Supplemental Figure 10). We also used an acute dose of indomethacin, an NSAID known to undergo enterohepatic recirculation, to address the possibility that the DKO-induced dysbiosis might influence bacterial glucuronidase activity to modify the duration of drug exposure (55, 60, 71) (Supplemental Figure 11A). However, no difference in glucuronidated versus parent indomethacin was observed between DKOs and controls (Supplemental Figure 11B).

**Figure 5:**
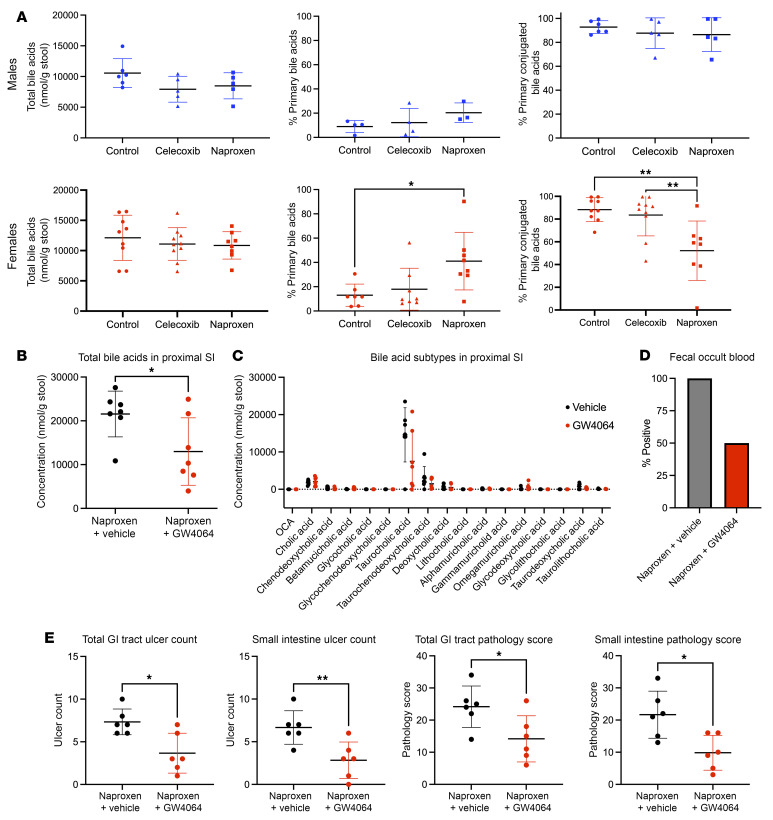
Distinct gut microbe–dependent bile acid conversions are observed with chronic naproxen treatment, and suppression of bile acid synthesis protects against naproxen-induced enteropathy in a COX-independent manner. (**A**) WT C57BL/6J mice were treated with either control diet, celecoxib diet (100 mg/kg), or naproxen diet (230 mg/kg) and allowed to feed ad libitum for 3 weeks prior to tissue collection. *n* = 5–9 mice per group. Bile acids (BAs) were measured in intestinal luminal content samples via UPLC. *P < 0.05, **P < 0.01 by 1-way ANOVA. (**B**–**E**) *Cox*-DKO mice were fed naproxen diet (230 mg/kg) ad libitum, paired with either 30 mg/kg GW4064 or 0.5% methylcellulose (vehicle) by twice daily oral gavage for 10 days. *n* = 6 mice per group, females only. (**B**) Total bile acids in proximal small intestine luminal contents measured by UPLC. *P < 0.05 by unpaired 1-tailed *t* test. (**C**) Individual bile acids in proximal small intestine luminal contents measured by UPLC. (**D**) Hemoccult test reported as percentage of mice that tested positive in each treatment group within the 10-day period. (**E**) Ulcer count for total GI tract versus small intestine only as well as pathology score for total GI tract versus small intestine only. *P < 0.05, **P < 0.01 by unpaired 1-tailed *t* test.

Naproxen also undergoes enterohepatic recirculation, and intestinal bacteria may influence bile acid conjugation; deconjugation of primary bile acids has been shown to increase their toxicity to intestinal mucosa (70, 72). We observed that naproxen exposure in WT C57BL/6 mice resulted in decreased bile acid conjugation (Figure 5A). Bile acids activate the farnesoid X receptor (FXR), which downregulates CYP7A1 and thereby the hepatic conversion of cholesterol to primary bile acids. Here, we showed that the FXR agonist GW4064 suppressed total bile acid levels in the proximal small intestinal lumen relative to vehicle (0.5% methylcellulose) on a *Cox*-DKO background (Figure 5B). This suppression was largely driven by the most abundant bile acid subtype, taurocholic acid (Figure 5C). We then demonstrated that coadministration of the FXR agonist with naproxen protected against naproxen-induced GI bleeding (Figure 5D) and reduced ulcer count and pathology score (Figure 5E), and this protective effect was predominantly seen in the small intestine.

We also validated microbiome-dependent bile acid patterns in a separate 16S study of C57BL/6 WT mice treated with naproxen that could help explain the observed sexual dimorphism. In this dataset, we identified 3 differentially abundant taxa in females on naproxen that were not differentially abundant in males: Firmicutes Clostridium, Firmicutes Lachnospiraceae, and Firmicutes Lachnoclostridium (Supplemental Figure 12, A and B). The first of these taxa yielded the same pattern and direction as for the lactobacilli seen in the DKOs; an increase in Firmicutes Clostridium results in increased bile salt hydrolase (73), which results in more deconjugation of primary bile acids specifically in female mice treated with naproxen. The other 2 differentially abundant taxa in the WT naproxen females do not possess bile salt hydrolase, but do possess 7α-dehydroxylase, which is responsible for converting primary bile acids to secondary bile acids; a decrease in Firmicutes Lachnospiraceae NK4A136 results in decreased bile acid 7α-dehydroxylation (74), which results in less conversion of primary bile acids to secondary bile acids. The same applies with the decrease in Firmicutes Lachnoclostridium (75). Both mechanisms could help explain the increase in the percentage of primary bile acids in the naproxen-treated females.

### Administration of NSAIDs and antibiotics together results in higher risk of GI bleeding in 2 separate human patient cohorts.

**Figure 6:**
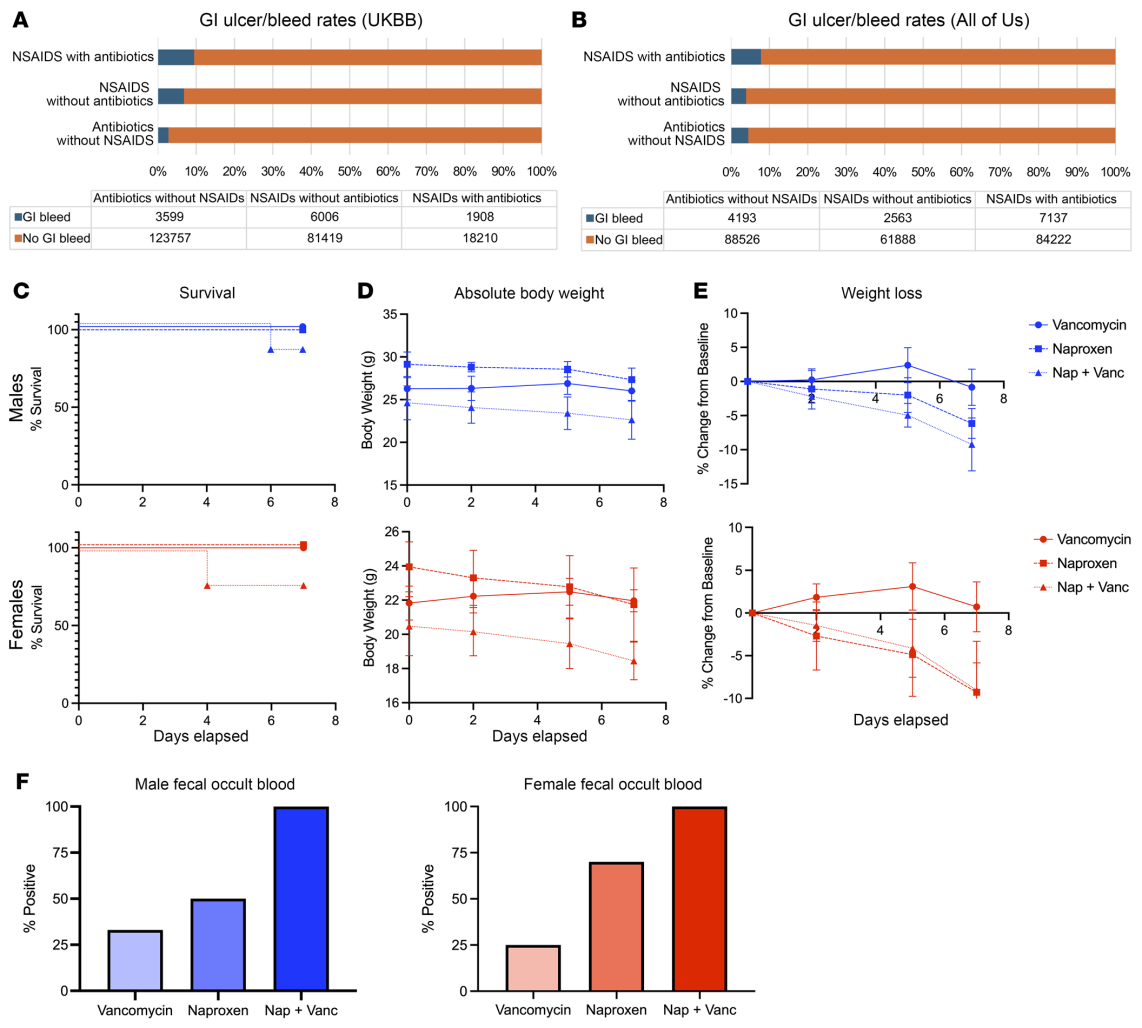
Coincident administration of NSAIDs and antibiotics in humans increases the risk of GI bleeding. Queries of GI ulceration and bleeding rates in human patients taking NSAIDs alone, antibiotics alone, or concurrent NSAIDs and antibiotics using UK Biobank (**A**) and All of Us (**B**). For the UK Biobank (UKBB) population, the relative incidence of GI bleeding for patients taking NSAIDs alone versus in combination with antibiotics was 6.87% versus 9.48% (*P* = 1.9 × 10^–35^); for the All of Us population, the relative incidence of GI bleeding for patients taking NSAIDs alone versus in combination with antibiotics was 4.10% versus 8.47% (*P* < 1.0 × 10^–100^). *P* values were calculated by Fisher’s exact test. (**C**–**F**) Validation of human NSAIDs with antibiotics GI bleeding patterns in mice. *Cox*-DKO mice were treated with either vancomycin (0.5 g/L dissolved in the drinking water) or control drinking water and fed either 1,323 ppm naproxen diet or control diet ad libitum for 1 week. *n* = 6–10 mice per group. (**C**) Survival plotted as a Kaplan-Meier curve. (**D**) Change in absolute BW over 1 week. (**E**) Percent change in BW relative to baseline BW over 1 week. (**F**) Hemoccult test reported as percentage of mice that tested positive in each treatment group within the 7-day period.

To address the possibility that the gut microbiome might influence NSAID-induced enteropathy in humans, we assessed the impact of coincident antibiotic treatment with NSAIDs on enteropathy in 2 human retrospective cohorts: the UK Biobank (76) and All of Us Research Program (77). In both data sets, the incidence of NSAID-associated GI bleeding was greater when they were coadministered with antibiotics, potentially implicating the ablation of protective commensal bacteria (68, 70) (Figure 6, A and B). For the UK Biobank population, the relative incidence of GI bleeding for patients taking NSAIDs alone versus in combination with antibiotics was 6.87% versus 9.48% (Figure 6A, *P* = 1.9 × 10^–35^). For the All of Us population, the relative incidence of GI bleeding for patients taking NSAIDs alone versus in combination with antibiotics was 4.10% versus 8.47% (Figure 6B, *P* < 1.0 × 10^–100^).

We then performed a validation study to determine whether we could replicate these human patterns in our *Cox-*DKO mouse model. We chose vancomycin as it targets Gram-positive bacteria and has been shown to shift the gut microbial composition toward a higher abundance of Gram-negative taxa, resulting in more severe naproxen-induced intestinal damage in rats (70). This is consistent with the baseline differences in the DKO microbiome relative to *Cre^–/–^* controls in Figure 4, as cohousing protects against naproxen-induced enteropathy by increasing the relative abundance of protective Gram-positive commensal bacteria in the DKOs. To push the taxonomic composition in a further Gram-negative direction, DKO mice were pretreated with either 0.5 g/L vancomycin dissolved in drinking water or untreated drinking water for 1 week. Then, baseline BW and feces were collected, and mice were further separated into naproxen diet or control diet treatment groups for an additional week. Several mice in the naproxen + vancomycin coadministration group did not survive the full week, contrary to naproxen or vancomycin treatment alone (Figure 6C). Furthermore, naproxen + vancomycin coadministration resulted in greater absolute (Figure 6D) and relative (Figure 6E) weight loss compared with the other 2 treatment groups. Naproxen + vancomycin coadministration also resulted in more mice with blood detected in the stool relative to naproxen or vancomycin treatment alone (Figure 6F). Despite baseline differences between human versus murine microbiome composition, this pattern of more severe NSAID-induced GI injury occurring with antibiotic coadministration is conserved across species.

## Discussion

Although multiple mechanisms have been implicated in NSAID-induced enteropathy, here, we make the surprising observation that suppression of PG formation alone is of minimal, if any, importance. Prolonged follow-up of mice depleted postnatally of both COXs showed they did not develop enteropathy despite PG suppression. Furthermore, both the COX inhibitor naproxen and its structural analog, PPA, devoid of COX inhibitory activity evoke enteropathy in these mice. Thus, the enteropathy evoked by clinical levels of naproxen exposure is largely independent of its ability to suppress PG production.

A second surprising observation was that although adding naproxen to COX-depleted mice does not further suppress PG production, the severity of the NSAID-induced enteropathy is markedly augmented when compared with its evocation in WT mice. Depletion or inhibition of the COX enzymes caused dysbiosis of the gut microbiome and, consistent with prior observations, we observed a relative increase in Gram-negative taxa in the DKOs compared with control mice. While such a shift in the composition of the gut microbiota might have multiple effects of relevance to the augmentation of naproxen-evoked enteropathy, we saw no impact on absorbed bacterial metabolites, host immune function, or drug metabolism.

Consistent with a previous report (70), we found that naproxen may have increased bile acid cytotoxicity by promoting the deconjugation of primary bile acids in WT mice. Thus, coadministration of an FXR agonist to the DKO mice significantly reduced the severity of naproxen-evoked enteropathy, as previously reported in WT mice (78). Bile duct ligation also successfully prevents the formation of indomethacin-induced enteric ulcers in mice (51), further implicating bile acids as an important contributor to NSAID-induced enteropathy. We acknowledge that bile acid pathways in mice differ from those in humans. However, there is much functional redundancy amongst bile acid–modifying enzymes that bacterial taxa can possess (e.g., bile salt hydrolases; refs. 73, 79).

To acquire clinical evidence connecting the dysbiosis caused by PG suppression with NSAID-evoked enteropathy, we compared its incidence in patients with and without concomitant treatment with antibiotics. The higher rates of enteropathy in the UK Biobank and All of Us cohorts on the drug combination are consistent with the hypothesis that microbial ablation by the antibiotics removed commensals that limit NSAID-evoked tissue injury, whether by topical irritation, bile acids, or other mechanisms. Postoperatively, patients often receive both treatments. A limitation is that these databases cannot identify the location of injury within the GI tract. Also, this study does not evaluate the effects of social or most demographic factors on the risk of NSAID-induced small intestinal damage.

Although naproxen-induced enteropathy appears more common and severe in females in the mice, this observation requires further investigation, given the sample sizes and very limited support in existing literature (52, 80, 81). Similarly, clinical trials of NSAIDs are heavily biased toward inclusion of women (82–84), and interrogation of a sex-dependent incidence of NSAID-induced enteropathy in humans has been similarly underpowered (85–88). Our work frames the hypothesis that differential abundance of key bacterial taxa and their corresponding bile acid–modifying enzymes may contribute to the sexual dimorphism that we observed.

In summary, PG suppression plays a trivial role in NSAID-induced enteropathy. However, *Cox* deletion causes dysbiosis of the gut microbiome that amplifies the enteropathic response to NSAIDs.

## Methods

### Sex as a biological variable

Both male and female mice and humans were included throughout all experiments and analyzed separately versus combined to account for sex as a biological variable.

### Animals

All mice were bred and maintained in our animal facility and fed ad libitum with standard chow diet (LabDiet 5010, Laboratory Autoclavable Rodent Diet) unless placed on a special formulated diet for a particular study. Mice were kept under a 12-hour light/12-hour dark cycle, with lights on at 7 am and lights off at 7 pm. Male and female mice aged 10–12 weeks were used for all experiments, and data were always analyzed separately for each sex.

### Genotypes

All mice were on a C57BL/6J background. *Cox-1^fl/fl^* mice (89) were gifted to us from the Herschman Lab at UCLA, and they were selectively bred with our tamoxifen-inducible *Cox-2^fx/fx^ CMV-Cre^+/–^* mice (90) that we maintained in-house to yield the *Cox-1^fl/fl^ Cox-2^fl/fl^ CMV-Cre^+/–^* and *Cox-1^fl/fl^ Cox-2^fl/fl^ CMV-Cre^–/–^* mice that were used throughout these experiments for universal *Cox*-DKO and *Cre^–/–^* controls derived from the same litter. Primer sequences for genotyping were as follows: *Cox-1* flox forward 1: 5′-TACCGCTGTCTCAGATTTTCCAGC-3′; *Cox-1* flox reverse 1: 5′-GTGGAGCTGAAGCTAGGAAACAGC-3′; *Cox-1* flox reverse 2: 5′-CCAGGCTTTACACTTTATGCTTCCG-3′; *Cox-2* flox forward: 5′-TGAGGCAGAAAGAGGTCCAGCCTT-3′; *Cox-2* flox reverse: 5′-ACCAATACTAGCTCAATAAGTGAC-3′; *CMV-Cre* forward: 5′-CGATGCAACGAGTGATGAGG-3′; *CMV-Cre* reverse: 5′-GCATTGCTGTCACTTGGTCGT-3′.

### Chemicals

All chemicals and materials were purchased from Sigma-Aldrich unless otherwise stated.

### Considerations for chronic NSAID dosing in mice

In humans, NSAID-induced enteropathy typically occurs after chronic NSAID exposure, as seen in patients with osteoarthritis or rheumatoid arthritis. To mimic this condition, we exposed mice to a 3-week naproxen-containing diet, during which they recapitulated many of the clinical features of NSAID-induced enteropathy observed in humans. After just 1 week of treatment, the mice began to show early signs of enteropathy, such as weight loss and positive fecal occult blood tests. We carefully dosed naproxen and celecoxib in mice to achieve therapeutic plasma concentrations relevant to humans. A diet containing 230 mg/kg of naproxen corresponds to a human equivalent dose (HED) of 18.7 mg/kg, while a diet containing 100 mg/kg of celecoxib corresponds to an HED of 8.1 mg/kg. The HEDs were calculated from the animal doses using the formula reported by Nair and Jacob (64).

### In vivo study designs

#### Study 1 design: longitudinal tracking of Cox-DKO mice.

Baseline weight, feces, and urine were collected (prior to Cre-recombinase activation by tamoxifen). Then mice of both *Cox*-DKO and *Cre^–/–^* control genotypes were administered tamoxifen (200 mg/kg BW per day for 5 consecutive days, dissolved in 85% corn oil/15% ethanol solution) via oral gavage at approximately 10–12 weeks of age to induce genetic deletion of *Cox-1* and *Cox-2*. One month was allowed for tamoxifen washout, then weight was measured and feces were collected weekly, and urine was collected monthly for 10 months (until the mice reached ~1 year of age). GI tissues were harvested and prepared for histology. *n* = 14 *Cox*-DKO mice and 6 *Cre^–/–^* controls.

#### Study 2 design: LPS challenge on Cox-DKO background.

Mice of both *Cox*-DKO and *Cre^–/–^* control genotypes were administered tamoxifen (200 mg/kg BW per day for 5 consecutive days, dissolved in 85% corn oil/15% ethanol solution) via oral gavage at approximately 10–12 weeks of age to induce genetic deletion of *Cox-1* and *Cox-2*. One month was allowed for tamoxifen washout. Then after baseline urine collection, mice received a single dose of LPS (1 mg/kg BW dissolved in 1× PBS) by i.p. injection, and urine was collected overnight in metabolic cages. *n* = 7-9 mice per group.

#### Study 3 design: pharmacokinetic time course of naproxen in WT mice.

Adult WT C57BL/6J mice were fed 1,323 ppm naproxen diet (Envigo) ad libitum for 1 week to achieve steady state. Then blood was collected from the retroorbital vein at the following time points after naproxen exposure: 0, 2, 4, 6, 8, and 16 hours. Plasma drug concentrations were then measured by liquid chromatography with tandem mass spectrometry (LC-MS/MS). *n* = 3–6 mice per time point, females only.

#### Study 4 design: chronic naproxen exposure on WT background.

Adult WT C57BL/6J mice were fed control diet (Purina Lab Chow, Envigo) ad libitum for 2 weeks, at which point baseline weight was measured and feces and urine were collected. Then cages were assigned either control diet or an identical diet with 1,323 ppm naproxen or 550 ppm celecoxib dry-mixed into the formulation (Naproxen Custom Diet, Envigo) for 3 weeks. Weight was measured and feces were collected each week, and urine was collected at the end of the 3-week period prior to tissue harvest. GI tissues were fixed for histology, and blood was collected for complete blood counts and serum protein measurements. *n* = 6–9 mice per group.

#### Study 5 design: chronic naproxen exposure on Cox-DKO background.

Mice of both *Cox*-DKO and *Cre^–/–^* control genotypes were administered tamoxifen (200 mg/kg BW per day for 5 consecutive days, dissolved in 85% corn oil/15% ethanol solution) via oral gavage at approximately 10–12 weeks of age to induce genetic deletion of *Cox-1* and *Cox-2*. One month was allowed for tamoxifen washout, during which mice were fed control diet (Purina Lab Chow) ad libitum. Then after baseline weight measurement and feces and urine collection, cages were assigned either control diet or an identical diet with 1,323 ppm naproxen dry-mixed into the formulation (Naproxen Custom Diet) for 3 weeks. Weight was measured and feces were collected each week, and urine was collected at the end of the 3-week period before tissues were harvested. GI tissues were fixed for histology, and blood was collected for complete blood counts. This experiment was performed 2 slightly different ways to assess relative contribution of the gut microbiome and host immune system: (a) littermate mice of both genotypes cohoused in the same cage for the duration of the experiment and (b) littermate mice separated by genotype prior to tamoxifen administration; this second version was terminated at 10 days rather than 21 days due to increased rate of weight loss in *Cox*-DKO + naproxen cages. *n* = 7–10 mice per group.

#### Study 6 design: chronic PPA exposure on Cox-DKO background.

Mice of both *Cox*-DKO and *Cre^–/–^* control genotypes were administered tamoxifen (200 mg/kg BW per day for 5 consecutive days, dissolved in 85% corn oil/15% ethanol solution) via oral gavage at approximately 10–12 weeks of age to induce genetic deletion of *Cox-1* and *Cox-2*. One month was allowed for tamoxifen washout. Then after baseline weight measurement and feces and urine collection, mice were fed a diet with 1,323 ppm PPA dry-mixed into the formulation (PPA Custom Diet, Envigo) for 3 weeks. Weight was measured and feces were collected each week, and urine was collected at the end of the 3-week period before tissues were harvested and prepared for histology. *n* = 6–9 mice per group.

#### Study 7 design: coadministration of FXR agonist and naproxen on Cox-DKO background.

*Cox-1^fl/fl^ Cox-2^fl/fl^ CMV-Cre^+/–^* mice were administered tamoxifen (200 mg/kg BW per day for 5 consecutive days, dissolved in 85% corn oil/15% ethanol solution) via oral gavage at approximately 10–12 weeks of age to induce genetic deletion of *Cox-1* and *Cox-2*. One month was allowed for tamoxifen washout. All mice were fed 1,323 ppm naproxen diet ad libitum, paired with either 30 mg/kg BW GW4064 (BePharm Scientific) or 0.5% methylcellulose (vehicle) by twice daily oral gavage. Feces, small intestine luminal contents, and GI tissue were collected after 10 days of treatment. *n* = 6 mice per group, females only.

#### Study 8 design: microbiome-dependent elimination of indomethacin.

Adult WT C57BL/6J mice were administered either an antibiotic cocktail (1 g/L ampicillin, 0.2 g/L vancomycin, 1 g/L neomycin, 1 g/L metronidazole, and 4 g/L aspartame) or vehicle (4 g/L aspartame) in their drinking water for 1 week prior to receiving a single dose of indomethacin (10 mg/kg BW dissolved in PEG400) via oral gavage, and urine was collected for the following 4 hours to measure indomethacin-glucuronide/indomethacin ratio. *n* = 4 mice per group. Additionally, separately housed *Cox-*DKO mice and Cre-negative controls were treated with a single dose of indomethacin (10 mg/kg BW dissolved in PEG400) via oral gavage, and urine was collected for the following 4 hours to measure urinary indomethacin-glucuronide/indomethacin ratio. *n* = 10–11 mice per group.

#### Study 9 design: coadministration of vancomycin and naproxen on Cox-DKO background.

*Cox-1^fl/fl^ Cox-2^fl/fl^ CMV-Cre^+/–^* mice were administered tamoxifen (200 mg/kg BW per day for 5 consecutive days, dissolved in 85% corn oil/15% ethanol solution) via oral gavage at approximately 10–12 weeks of age to induce genetic deletion of *Cox-1* and *Cox-2*. One month was allowed for tamoxifen washout. Then mice were treated with either vancomycin (0.5 g/L dissolved in the drinking water) or control drinking water for 1 week. After this pretreatment, baseline weight was measured and feces were collected. Then mice were fed either 1,323 ppm naproxen diet or control diet (Purina Lab Chow) ad libitum for 1 week, weight was measured, and feces were collected at days 2, 5, and 7. *n* = 6–10 mice per group.

### MS analysis of prostanoids

Urinary PG metabolites were measured by LC-MS/MS as previously described (91). Briefly, urine samples were collected using metabolic cages over 4-hour periods. Labeled internal standards of known concentrations for metabolites of PGE_2_, PGD_2_, PGI_2_, and TxB_2_ were spiked into urine samples prior to solid phase extraction (SPE). Then SPE was performed using Strata-X 33 μm polymeric reversed phase cartridges (Phenomenex). Spectra were generated using a Waters ACQUITY UPLC system. Peak area ratios of target analytes to internal standards were calculated using TargetLynx 4.1 software (Waters). Urinary prostanoid metabolite measurements were normalized to urinary creatinine measurements.

### Histological analysis of GI injury

All histology sectioning, staining, and analysis were performed at the Penn Vet Comparative Pathology Core (RRID:SCR_022438). Briefly, during dissection, the stomach was isolated, and intestinal tissues were separated into 4 segments: proximal small intestine (~duodenum), middle small intestine (~jejunum), distal small intestine (~ileum), and colon. The stomach was first injected with fixative (methacarn solution: 60% methanol, 30% chloroform, and 10% acetic acid) to preserve its shape, placed into a 50 mL conical tube filled with fixative, and later transferred to a cassette. Intestinal segments were Swiss rolled, placed into separate 50 mL conical tubes filled with fixative, and later transferred to cassettes. Samples were then transported to the core facility to be dehydrated with ethanol, embedded in paraffin, cut into 5 μm sections, and stained with H&E. Slides were then evaluated by a trained, fully blinded veterinary pathologist with no knowledge of respective treatment or genotype identities among the samples. The qualitative/semiquantitative scoring system included assessment of ulcer distribution (none, focal, or multifocal), number of ulcers, ulcer severity (superficial, up to muscularis mucosa, or transmural), presence of peritonitis/serositis, and inflammation (none, minimal, mild, moderate, or severe).

### Fecal occult blood test

The Hemoccult Sensa Rapid Test Kit (Beckman Coulter) was used to detect blood in freshly collected stool samples on a weekly basis. Briefly, if hemoglobin is present in a fecal sample, the resulting peroxidase activity catalyzes the oxidation of α-guaiaconic acid (in the test strip) by hydrogen peroxide (in the developer solution) to form a conjugated blue quinone compound. If the test strip turns blue, then the fecal sample is positive for occult blood. Results were reported as positive or negative (not quantifiable beyond binary designation).

### Complete blood count analysis

Whole blood (>100 μL per sample) was collected in K_2_EDTA-coated tubes to prevent clotting and briefly stored at room temperature. Samples were then immediately transported to the CHOP Translational Core Laboratory within 3–4 hours of collection to perform complete blood count (CBC) analysis using a Sysmex XT-2000iV automated hematology analyzer (Sysmex America).

### Serum protein measurements

The serum proteins and liver enzymes were measured by the CHOP Translational Core using the Roche Cobas c311 platform, a clinical automated chemistry analyzer.

### Fecal calprotectin measurements

Calprotectin was measured in murine stool samples. The samples were homogenized and analyzed according to the manufacturer’s instructions (100A8/A9/Calprotectin ELISA, Immunodiagnostik). Results were expressed as nanograms of calprotectin per microgram of sample.

### 16S rRNA gene sequencing

#### Library preparation (16S V1–V2 high biomass).

Bacterial DNA was isolated from fecal and small intestine luminal content samples via the DNeasy PowerSoil Pro Kit (Qiagen). Isolated samples were then submitted to the Penn CHOP Microbiome Core for all subsequent steps. Barcoded PCR primers annealing to the V1–V2 region of the 16S rRNA gene were used for library generation. PCR reactions were carried out in duplicate using Q5 High-Fidelity DNA Polymerase (NEB). Each PCR reaction contained 0.5 μM each primer, 0.34 U Q5 polymerase, 1× buffer, 0.2 mM dNTPs, and 5 μL DNA in a total volume of 50 μL. Cycling conditions were as follows: 1 cycle of 98°C for 1 minute; 20 cycles of 98°C for 10 seconds, 56°C for 20 seconds, and 72°C for 20 seconds; 1 cycle of 72°C for 8 minutes. After amplification, duplicate PCR reactions were pooled and purified using a 1:1 volume of SPRI beads. DNA in each sample was then quantified using PicoGreen and pooled in equal molar amounts. The resulting library was sequenced on the Illumina MiSeq using 2x250 bp chemistry. Extraction blanks and DNA-free water were subjected to the same amplification and purification procedure to allow for empirical assessment of environmental and reagent contamination. Positive controls, consisting of 8 artificial 16S gene fragments synthesized in gene blocks and combined in known abundances, were also included.

#### Bioinformatics processing.

QIIME2 version 2023.2.0 (92) was employed to process sequencing reads with DADA2 version 1.26.0 (93) to denoise reads and identify amplicon sequence variants (ASVs). ASVs were assigned to taxonomies by sequence comparison to the SILVA 138 database (94) using a Naive Bayes classifier implemented in scikit-bio (95). MAFFT (96) was used to build a phylogenetic tree for calculating UniFrac distances (97, 98).

#### Statistical analyses.

Statistical analyses of ASV tables and diversity metrics were performed with R libraries. UniFrac distances were visualized with principal coordinate analysis plots, and differences between study groups were assessed using permutational multivariate ANOVA (PERMANOVA) (99) with the adonisplus R package, which utilizes the vegan adonis2 software. PERMANOVAs were run with 999 permutations, and cage effect was accounted for by restricted shuffling of samples between cages. Differential abundance of any taxon with an average abundance of at least 0.1% across all fecal samples was assessed by generalized linear mixed effects models on log_10_-transformed relative abundances. Cage number was included as a random effect and genotype as a fixed effect. Multiple tests were adjusted for FDR using the Benjamini-Hochberg method.

### Bile acid measurements

Bile acid quantifications were performed by the Microbial Culture and Metabolomics Core of the Penn CHOP Microbiome Program and the Center for Molecular Studies in Digestive and Liver Diseases as described previously (100, 101). Briefly, luminal contents were collected neat into preweighed tubes from the proximal half of the small intestine, and each individual sample weight was recorded (between 25 and 100 mg per sample). Samples were stored at –80°C. Samples and sample weights were then submitted to the Microbial Culture & Metabolomics Core, and 16 different bile acids were quantified in each sample using a Waters ACQUITY ultraperformance liquid chromatography (UPLC) system with a Cortecs UPLC C-18+ 1.6 μm 2.1 × 50 mm column and a QDa single quadrupole mass detector. Samples were suspended in methanol (5 μL/mg stool), vortexed for 1 minute, and centrifuged twice at 13,000*g* for 5 minutes. Intestinal flushes were vortexed for 1 minute and centrifuged twice at 13,000*g* for 5 minutes. The supernatant was transferred to a new tube, sealed, and stored at 4°C until analysis. The flow rate was 0.8 mL/min, the injection volume was 4 μL, the column temperature was 30°C, the sample temperature was 4°C, and the run time was 4 minutes per sample. Eluent A was 0.1% formic acid in water; eluent B was 0.1% formic acid in acetonitrile; the weak needle wash was 0.1% formic acid in water; the strong needle wash was 0.1% formic acid in acetonitrile; and the seal wash was 10% acetonitrile in water. The gradient was as follows: initial flow 70% eluent A; linear gradient to 100% eluent B over 2.5 minutes; hold at 100% eluent B for 0.6 minutes; and linear gradient to 70% eluent A over 0.9 minutes. The mass detection channels were as follows: +357.35 for chenodeoxycholic acid and deoxycholic acid; +359.25 for lithocholic acid; –407.5 for cholic, α-muricholic, β-muricholic, γ-muricholic, and ω-muricholic acids; –432.5 for glycolithocholic acid; –448.5 for glycochenodeoxycholic and glycodeoxycholic acids; –464.5 for glycocholic acid; –482.5 for taurolithocholic acid; –498.5 for taurochenodeoxycholic and taurodeoxycholic acids; and –514.4 for taurocholic acid. Samples were quantified against standard curves of at least 5 points and run in triplicate (chemicals obtained from Santa Cruz Biotechnology and Steraloids). Standard curves were run at the beginning and end of each metabolomics run. Blanks and standards were run every 8 samples. Measurements were normalized to luminal content sample weights.

### NSAIDs/antibiotics queries in human databases

To study rates of GI bleeding following NSAID treatment and how those rates differ with concomitant antibiotic use in a real patient population, we created lists of NSAIDs and antibiotics that we queried in both the UK Biobank (76) and All of Us (77) databases. We defined patients taking concomitant NSAIDs and antibiotics as those taking antibiotics within 5 days of being prescribed an NSAID, filtering out prescriptions for topical antibiotics. We then compared rates of a composite GI phenotype as defined by 1 or more instances of peptic ulcer, gastrojejunal ulcer, gastric ulcer, or duodenal ulcer in the medical records of those who did and did not receive antibiotics. We also created a list of antibiotics that specifically target Gram-positive and -negative bacteria and compared rates of GI bleeding between those classes of antibiotics as well. All *P* values were calculated by Fisher’s exact test.

Further information may be found in Supplemental Methods.

### Statistics

All statistical analyses were performed using GraphPad Prism version 9/10, QIIME/R Studio (for 16S sequencing analyses), or SciPy/Jupyter Notebook (for analyses in All of Us and UK Biobank). One-way ANOVA (*P* < 0.05 considered significant), unpaired 1-tailed *t* test (*P* < 0.05 considered significant), Fisher’s exact test, PERMANOVA, orthogonal partial least squares–discriminant analysis, and accompanying methods of FDR correction were all conducted as described in each figure legend. Wherever applicable, all data were plotted as mean ± SD.

### Study approval

Experimental protocols were reviewed and approved by the Institute for Animal Care and Use Committee at the University of Pennsylvania under protocol number 804445. The UK Biobank research has been conducted using the UK Biobank Resource under application number 32133.

### Data availability

All underlying data values can be found in the Supporting Data Values file. The 16S sequencing data can be accessed through the following link in the Sequence Read Archive (SRP655337): https://www.ncbi.nlm.nih.gov/sra?term=SRP655337

## Author contributions

KB wrote the manuscript, generated the *Cox*-DKO mouse line, designed/executed all in vivo experiments involving the transgenic mice, and directed collaborations regarding histology, flow cytometry, 16S sequencing, bile acids, metabolomics, and the human data. SG, USD, and RJ conducted all LC-MS/MS analyses. CH conducted the flow cytometry experiments. CAA performed all GI pathology scoring assessments. CT and NW conducted all 16S sequencing analyses. RL conducted all Western blots. AM completed the analyses derived from the Human Pan-GI Cell Atlas. LR performed the FXR agonist platelet activation studies. KK performed queries in UK Biobank and All of Us. AS conducted all NMR analyses. EF performed all bile acid quantifications and analyses. MDR, KB, AW, KC, FDB, and GDW oversaw the above experiments, provided reagents, and edited the manuscript. GAF supervised the project, acquired funding, and edited the manuscript. ER generated the chronic NSAID dosing model, supervised the project, acquired funding, and edited the manuscript.

## Funding support

This work is the result of NIH funding, in part, and is subject to the NIH Public Access Policy. Through acceptance of this federal funding, the NIH has been given a right to make the work publicly available in PubMed Central.

- NIH grant U54TR001878 to GAF.
- PhRMA Research Starter Grant in Translational Medicine and Therapeutics to ER.
- Merit Award from the American Heart Association to GAF.
- Microbial Culture and Metabolomics Core of the Center for Molecular Studies in Digestive and Liver Diseases (NIH P30DK050306) for bile acid work to GDW.
- Abramson Cancer Center Support grant NIH P30 CA016520 to CAA.
- NIH grant F31 HG013246 to KK.
- NIH grant R01-DK-134575 to MDR.

## Supplementary Material

Supplemental data

Unedited blot and gel images

Supporting data values

## Figures and Tables

**Table 1:**
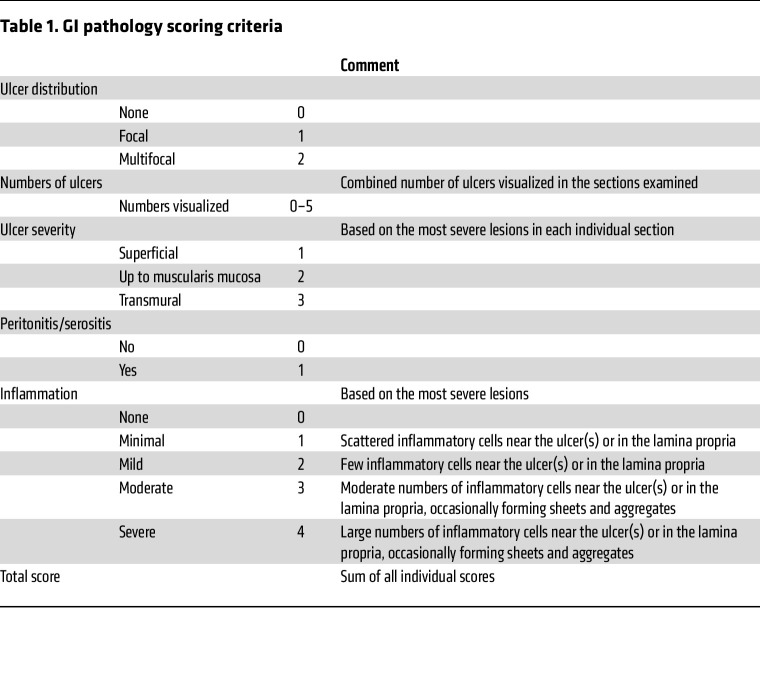
GI pathology scoring criteria

## Version 1 01/27/2026

In-Press Preview

## Version 2 03/16/2026

Electronic publication
