# Supplementary material for: Concomitant COX-1 and COX-2 suppression is not sufficient to induce enteropathy associated with chronic NSAID use
Source: J Clin Invest. 2026 Jan 27;136(6):e190575. doi: 10.1172/JCI190575 (PMC12987628; doi:10.1172/JCI190575)
Supplement: Supplemental data [file jci-136-190575-s059.pdf]

Supplemental Figure 1: COX-1 and COX-2 are expressed in human epithelial cells. Analysis of H5AD file downloaded from the Gut Cell Survey Atlas, filtered to include only small intestinal epithelial cells belonging to donors aged between 18 and 74 years, which yielded 193,038 cells in total. (**A**) Expression of COX-1 (PTGS1) across various intestinal epithelial cell subtypes and across four conditions: non-pathological, neighboring inflamed, Crohn's disease, and neighboring cancer. (**B**) Expression of COX-2 (PTGS2) across various intestinal epithelial cell subtypes and across the same four conditions. Plots only showing percentages of cells expressing PTGS1 or PTGS2. Microfold, INFLARE, and mucous gland neck cells were excluded due to low counts.

Supplemental Figure 2: Confirmation of suppressed gene expression in lung and small intestine of inducible *Cox*-DKO mice. *Cox*-DKO mice and $Cre^{-/-}$ control littermates received a single dose of LPS (1 mg/kg body weight dissolved in 1X PBS) or vehicle (1X PBS) by IP injection. Urine was collected overnight, and tissues were collected 12 hours after LPS administration. n = 4-7 mice per group, males only. (**A**) Gene expression via qPCR for *Ptgs1* and *Ptgs2*, which encode COX-1 and COX-2, respectively, in both lung and small intestine tissue. **p<0.01, ***p<0.001, ****p<0.0001 by unpaired one-tailed t test. Western blots and densitometry analysis of COX-1 protein levels in jejunal tissue unchallenged (**B**), COX-2 protein levels in jejunal tissue unchallenged (**C**), COX-1 protein levels in jejunal tissue challenged with LPS (**D**), and COX-2 protein levels in jejunal tissue challenged with LPS (**E**). *p<0.05 by unpaired one-tailed t test.

Supplemental Figure 3: Representative small intestine histology images as scoring examples. H&E-stained murine small intestine samples collected after 3 weeks of naproxen diet. Representative pictures of ulcer severity (**A**) and inflammation (**B**) displayed increasing from top to bottom. Note that the examples for “Ulcer Severity: 3 – Transmural” and “Inflammation: 4 – Severe” are derived from the same sample.

Supplemental Figure 4: Confirmation of plasma drug concentrations in chronic NSAID dosing model. Wildtype C57BL/6J mice were treated with either control diet, celecoxib diet (100 mg/kg), or naproxen diet (230 mg/kg) and allowed to feed *ad libitum* for 3 weeks prior to tissue collection. n = 6-9 mice

per group. (**A**) Plasma drug concentrations of celecoxib and naproxen measured by LC-MS/MS.

Supplemental Figure 5: Mice treated with naproxen exhibit a significant reduction in serum TxB_2, and the half-life of naproxen in mice is approximately 7.5 hours. (**A**) Serum TxB_2 measured by LC-MS/MS after 3-week treatment with control diet, naproxen diet (230 mg/kg), or celecoxib diet (100 mg/kg) on wildtype C57BL/6J background. n = 9-10 mice per group, females only. ****p<0.0001 by one-way ANOVA. (**B**) Pharmacokinetic time course of naproxen in wildtype C57BL/6J mice after treatment with naproxen diet (230 mg/kg) for one week to achieve steady state. Then blood was collected from the retroorbital vein at 0 hours, 2 hours, 4 hours, 6 hours, 8 hours, and 16 hours after termination of naproxen exposure. Plasma drug concentrations were then measured by LC-MS/MS. n = 3-6 mice per time point, females only.

Supplemental Figure 6: *Cox*-DKO mice develop gastrointestinal bleeding and lesions upon exposure to NSAID-analog phenylpropionic acid. *Cox*-DKO and *Cre*$^{-/-}$ control mice were treated with phenylpropionic acid diet (230 mg/kg) and allowed to feed *ad libitum* for 3 weeks prior to tissue collection. n = 6-9 mice per group for entire figure. (**A**) Urinary prostaglandin metabolites measured by LC-MS/MS. *p<0.05, **p<0.01, ****p<0.0001 by unpaired one-tailed t test. (**B**) Weekly hemoccult test results plotted as a KaplanMeier curve for percentage of each group that tested negative for blood in the stool. Any individual that tested positive would be marked positive for that first week and all subsequent weeks, resembling a survival curve. (**C**) Pathology score for total GI tract, small intestine alone, and stomach alone. *p<0.05, **p<0.01, ***p<0.001 by unpaired one-tailed t test. (**D**) Percent change in body weight relative to baseline body weight.

Supplemental Figure 7: Distinct microbiome composition in *Cox*-DKO mice is masked by cohousing. OTU-level differential abundance at baseline for separately housed animals (**A**) and cohoused animals (**B**). n = 7-10 mice per group for entire figure. Differential abundance of any taxon with an average abundance of at least 0.1% across all fecal samples was assessed by generalized linear mixed effects models on log10-transformed relative abundances. Multiple tests were adjusted for false discovery rate

(FDR) using the Benjamini-Hochberg method. Any taxon with an FDR < 0.1 is displayed in bold text.

Supplemental Figure 8. *Lactobacillus murinus, Bacteroides uniformus, Bacteroides acidifaciens,* and an uncultured *Bacteroides* species were more abundant in *Cox*-DKO mice when separately housed by genotype, but no statistically significant differences were detected at species level when cohoused. Differential abundance at baseline for separately housed animals (**A**-**B**) and cohoused animals (**C**) using species level calls of 16S data. n = 7-10 mice per group for entire figure. Linear mixed effects models were used to estimate the change in relative abundance of select taxa between study groups. The relative abundances were log10 transformed. Multiple tests were adjusted for false discovery rate using Benjamini-Hochberg method. Only the terms with p<0.1 are shown in the tables. Plotted are the taxa with q<0.1 and any taxa that were in *Turicibacter, Dubosiella, Alistipes, or Prevotellaceae.* The names containing "s__" indicate the ASVs that could be classified to species-level.

Supplemental Figure 9: Targeted and untargeted metabolomics revealed no distinct fecal bacteria-derived metabolites of interest, apart from low glucose in the *Cox*-DKO mice treated with naproxen. Separately housed *Cox*-DKO and $Cre^{-/-}$ control mice were treated with either control diet or naproxen diet (230 mg/kg) and allowed to feed *ad libitum* for 10 days prior to tissue collection. n=5-7 mice per group for entire figure. (**A**) Measurements of fecal short-chain fatty acids (acetate, butyrate, and propionate) via targeted NMR. (**B**) Principal Component Analysis plots of fecal metabolites clustered by treatment group via untargeted NMR, paired with supervised Orthogonal Partial Least Square – Discriminant Analysis (OPLS-DA). *p<0.05 by CV-ANOVA.

Supplemental Figure 10: Divergent gut microbiome composition did not result in altered immune cell populations in *Cox*-DKOs at baseline. Separately housed *Cox*-DKO and $Cre^{-/-}$ control mice were treated with either control diet or naproxen diet (230 mg/kg) and allowed to feed *ad libitum* for 10 days prior to tissue collection. n=5-7 mice per group for entire figure. (**A**) Representative flow cytometry plots for monocytes and neutrophils. Heatmap summary of flow cytometry for spleen (**B**) and blood (**C**). Individual

data points plotted for spleen monocytes (**D**) and spleen neutrophils (**E**). *p<0.05, **p<0.01, ***p<0.001 by one-way ANOVA.

Supplemental Figure 11: Baseline differences in microbiome composition between *Cox*-DKOs and controls did not result in differential drug elimination kinetics via deglucuronidation. (**A**) Wildtype C57BL/6J mice were administered either an antibiotic cocktail (1 g/L ampicillin, 0.2 g/L vancomycin, 1 g/L neomycin, 1 g/L metronidazole, and 4 g/L aspartame) or vehicle (4 g/L aspartame) in their drinking water for one week prior to receiving a single dose of indomethacin (10 mg/kg bodyweight dissolved in PEG400) via oral gavage, and urine was collected for the following 4 hours. Urinary indomethacin-glucuronide / indomethacin ratio measured by LC-MS/MS. n = 4 mice per group. ***p<0.001 by unpaired one-tailed t test. (**B**) Separately housed *Cox*-DKO and *Cre*$^{-/-}$ control mice were treated with a single dose of indomethacin (10 mg/kg bodyweight dissolved in PEG400) via oral gavage, and urine was collected for the following 4 hours. Urinary indomethacin-glucuronide / indomethacin ratio measured by LC-MS/MS. n = 10-11 mice per group.

Supplemental Figure 12. Separate validation cohort of C57/BL6 mice treated with naproxen produces shift in key taxa resulting in same pattern of increased primary bile acids that was observed in naproxen-treated female *Cox*-DKO mice. Wildtype C57BL/6J mice were treated with either control diet, celecoxib diet (100 mg/kg), or naproxen diet (230 mg/kg) and allowed to feed *ad libitum* for 3 weeks. Fecal pellets were collected at baseline and at the end of the three-week period for 16S sequencing.

n = 9-10 mice per group, females only. (**A**) Boxplots of differentially abundant taxa at OTU level for naproxen treatment group between Day 0 and Day 21. (**B**) Boxplot of differentially abundant taxon at ASV level for naproxen treatment group between Day 0 and Day 21. Differential abundance of any taxon with an average abundance of at least 0.1% across all fecal samples was assessed by generalized linear mixed effects models on log10-transformed relative abundances. Multiple tests were adjusted for false discovery rate (FDR) using the Benjamini-Hochberg method.

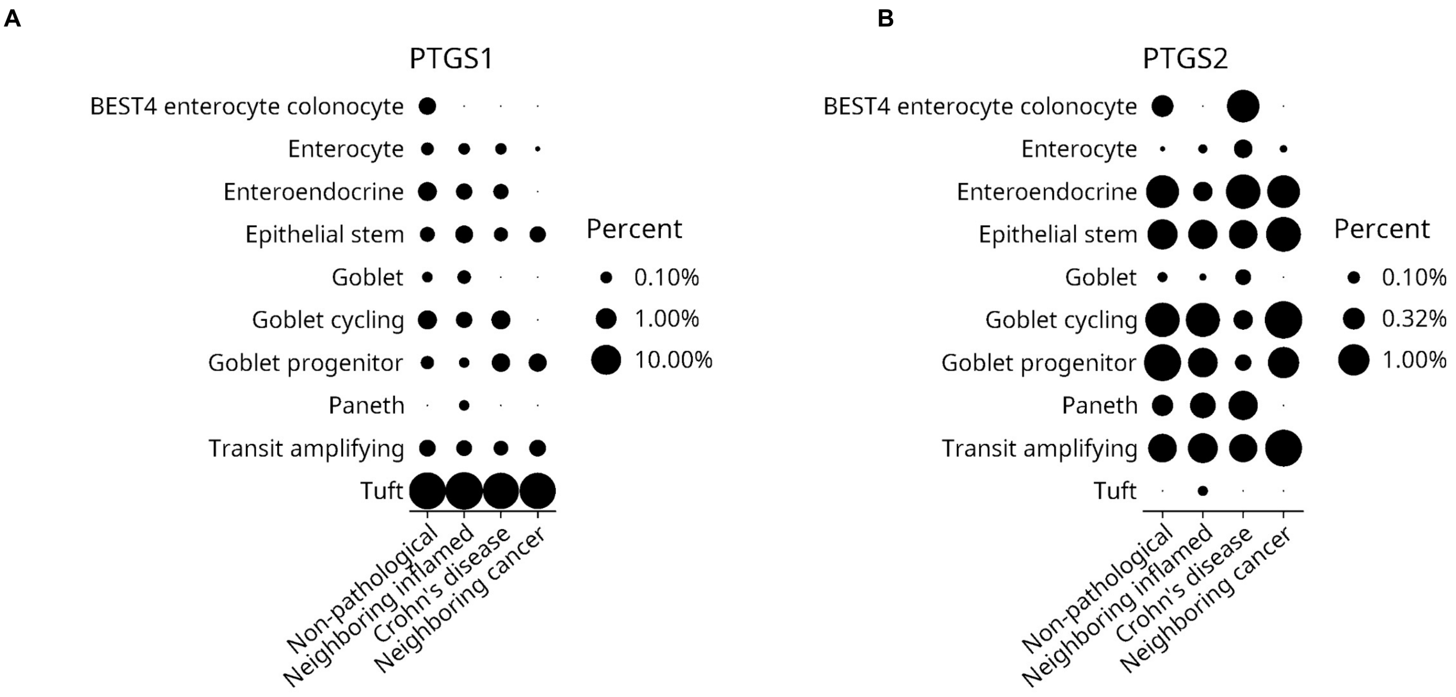

Supplemental Figure 1

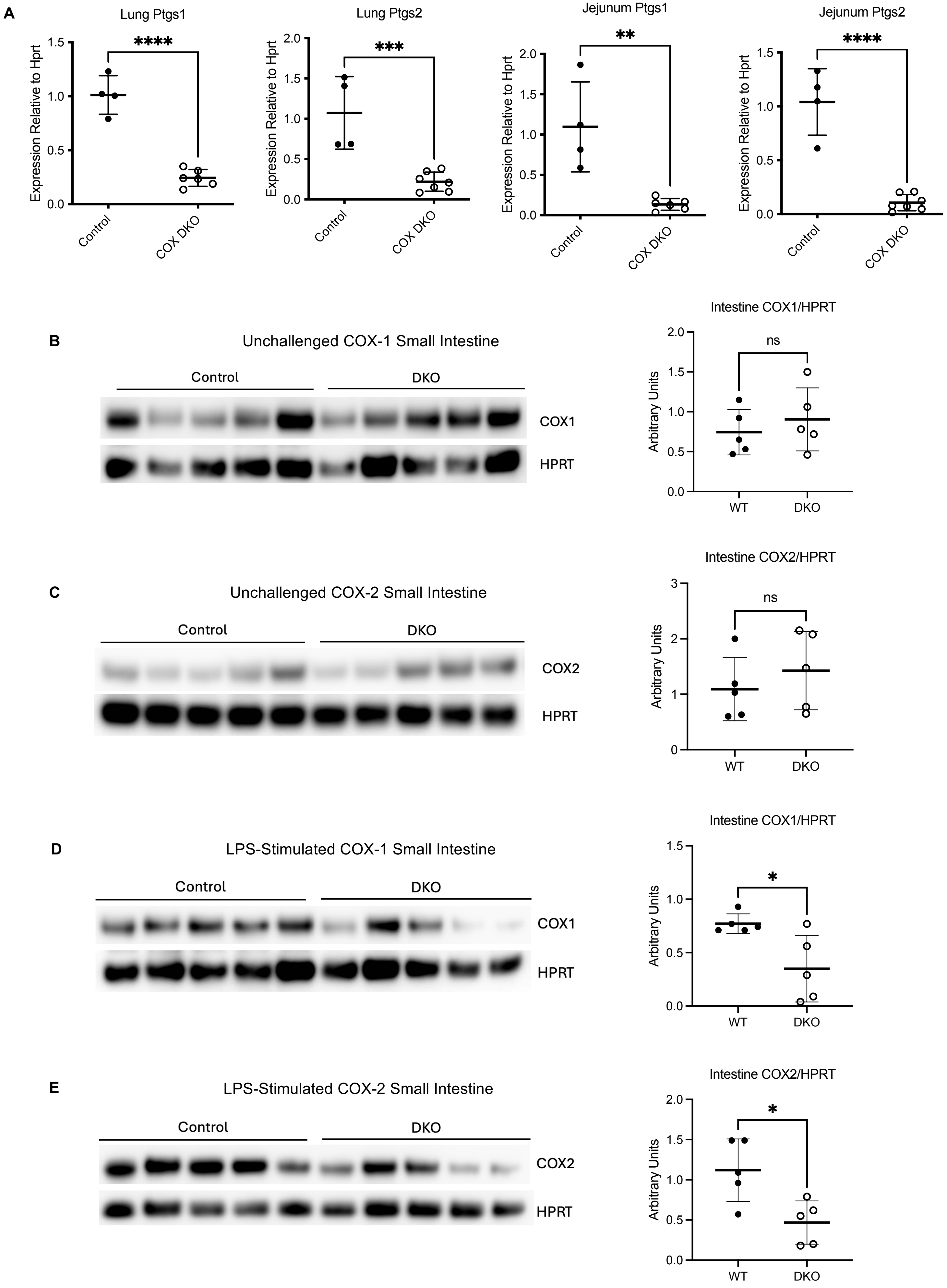

Supplemental Figure 2

A

Ulcer Severity

0 – No Ulcer / No Inflammation

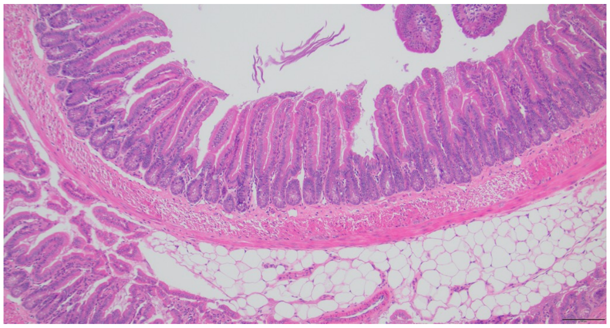

1 – Superficial

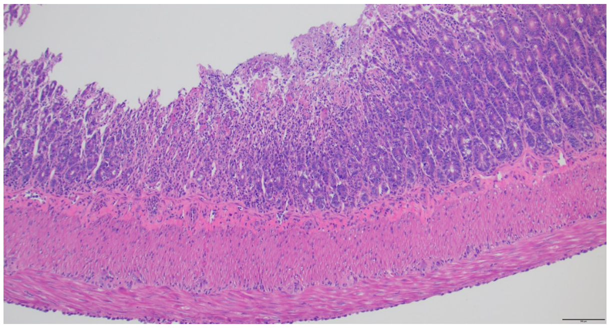

2 – Up to Muscularis Mucosa

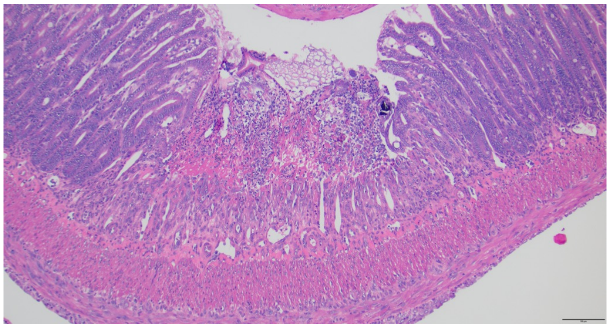

3 – Transmural

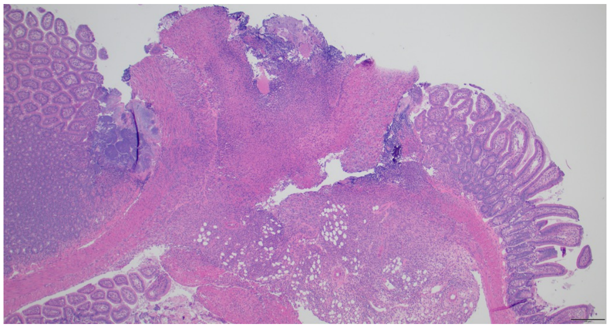

B

Inflammation

1 – Minimal

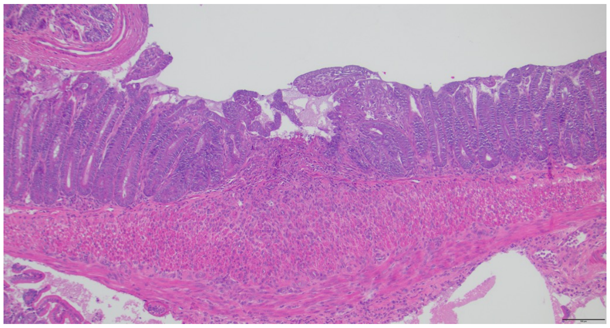

2 – Mild

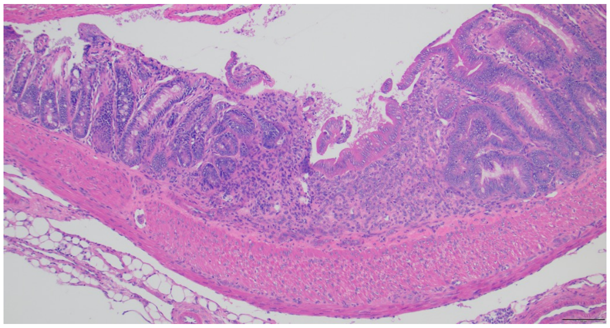

3 – Moderate

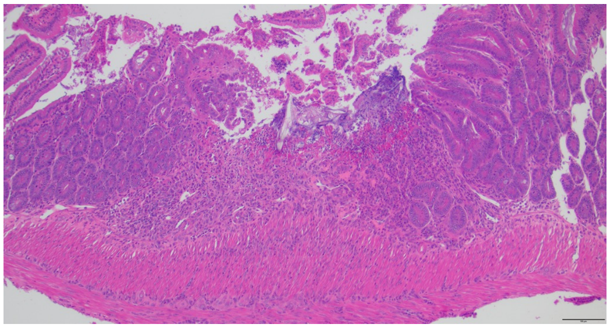

4 – Severe

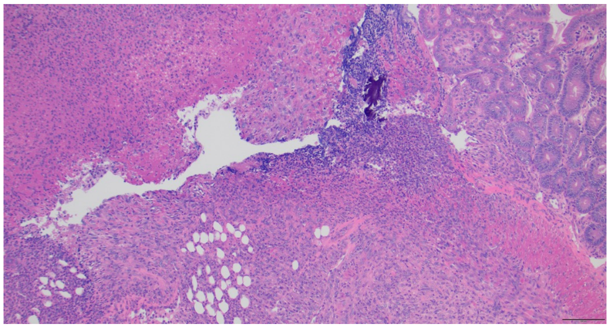

Supplemental Figure 3

A

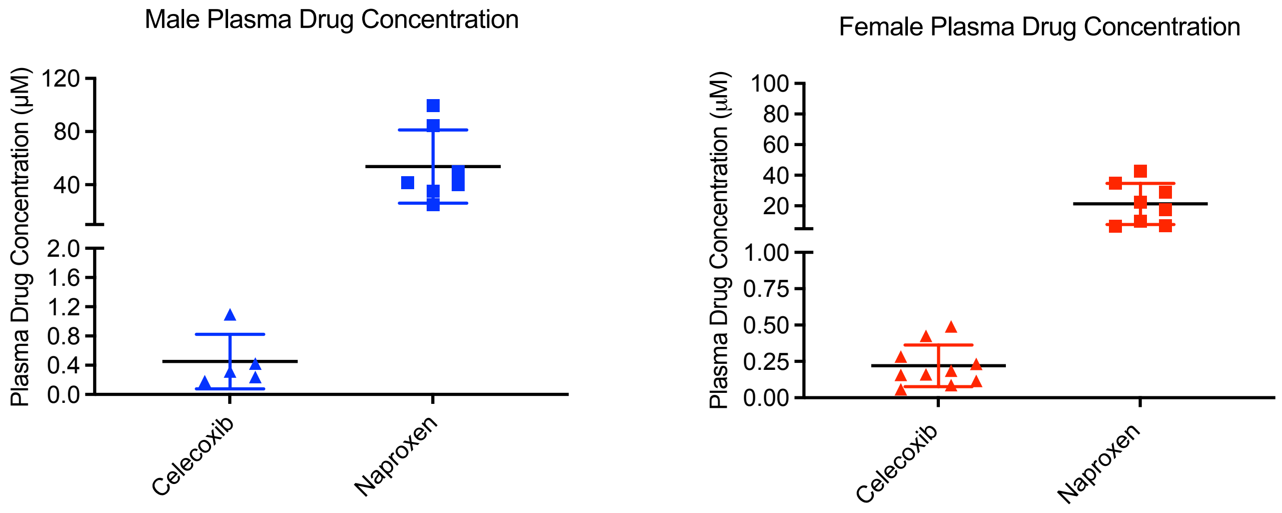
Male Plasma Drug Concentration
Plasma Drug Concentration (µM)
120
80
40
2.0
1.6
1.2
0.8
0.4
0.0
Celecoxib
Naproxen
Female Plasma Drug Concentration
Plasma Drug Concentration (µM)
100
80
60
40
20
1.00
0.75
0.50
0.25
0.00
Celecoxib
Naproxen

A

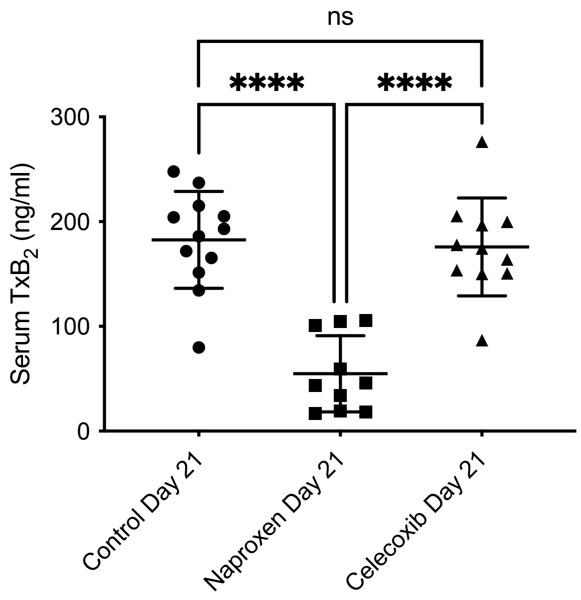

B

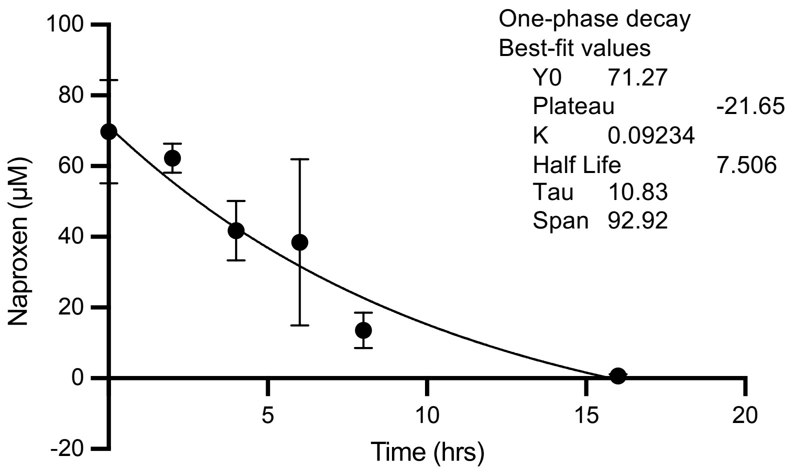

Supplemental Figure 5

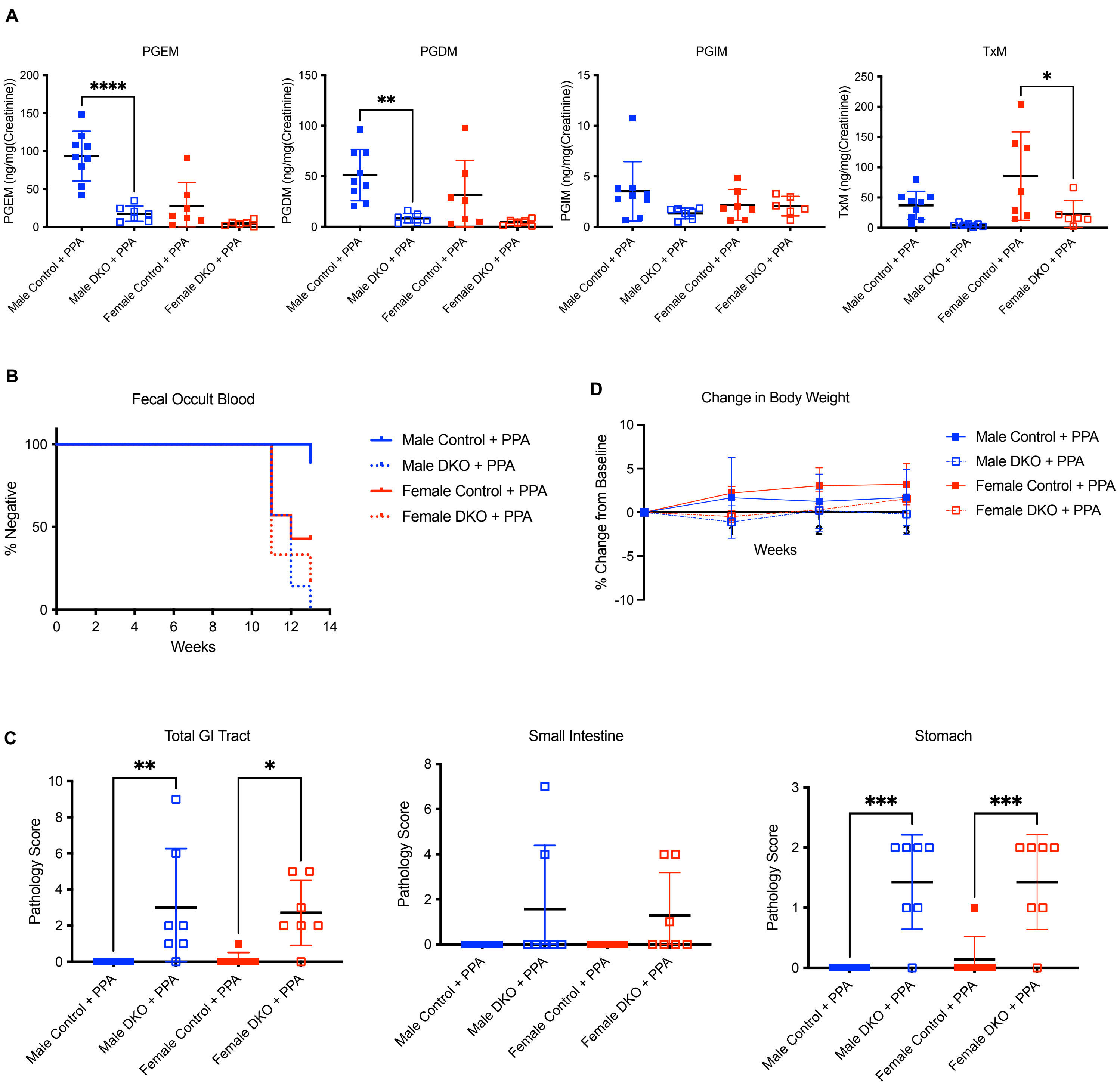

Supplemental Figure 6

A

Separately Housed OTU-Level Differential Abundance at Baseline

sample_type	sex	term	effect	Taxa	estimate	std.error	df	statistic	p.value	fdr
feces	female	WT v COX.DKO	fixed	p Bacteroidota f Prevotellaceae	0.37	0.11	14	3.42	0.00	**0.044**
	female	WT v COX.DKO	fixed	p Bacteroidota g Alistipes	0.42	0.15	14	2.77	0.02	**0.099**
	female	WT v COX.DKO	fixed	p Bacteroidota g Bacteroides	0.59	0.17	14	3.49	0.00	**0.044**
	female	WT v COX.DKO	fixed	p Bacteroidota g Prevotellaceae UCG-001	0.44	0.15	14	3.03	0.01	**0.067**
	female	WT v COX.DKO	fixed	p Firmicutes g Lactobacillus	0.58	0.19	14	3.13	0.01	**0.06**
	female	WT v COX.DKO	fixed	p Firmicutes g Turicibacter	-2.91	0.82	14	-3.52	0.00	**0.044**
	male	WT v COX.DKO	fixed	p Bacteroidota f Prevotellaceae	0.24	0.08	14	3.14	0.01	**0.06**
	male	WT v COX.DKO	fixed	p Bacteroidota g Alistipes	0.83	0.24	14	3.42	0.00	**0.044**
	male	WT v COX.DKO	fixed	p Bacteroidota g Muribaculum	-0.22	0.09	14	-2.39	0.03	0.17
	male	WT v COX.DKO	fixed	p Bacteroidota g Odoribacter	0.51	0.20	14	2.52	0.02	0.14
	male	WT v COX.DKO	fixed	p Firmicutes g Clostridia vadinBB60 group	0.72	0.20	14	3.57	0.00	**0.044**
	male	WT v COX.DKO	fixed	p Firmicutes g Dubosiella	-2.57	0.72	14	-3.59	0.00	**0.044**
	male	WT v COX.DKO	fixed	p Firmicutes g Faecalibaculum	-2.36	0.45	14	-5.19	0.00	**0.01**
	male	WT v COX.DKO	fixed	p Firmicutes g Lachnoclostridium	0.56	0.20	14	2.73	0.02	**0.099**
	male	WT v COX.DKO	fixed	p Firmicutes g Turicibacter	-2.15	0.91	14	-2.35	0.03	0.17

B

Co-Housed OTU-Level Differential Abundance at Baseline

sample_type	sex	term	effect	Taxa	estimate	std.error	df	statistic	p.value	fdr
Feces	female	WT v COX.DKO	fixed	p Bacteroidota f Prevotellaceae	0.29	0.13	12	2.26	0.04	0.44
	female	WT v COX.DKO	fixed	p Bacteroidota g Bacteroides	0.40	0.13	12	3.02	0.01	0.25
	female	WT v COX.DKO	fixed	p Bacteroidota g Rikenella	0.65	0.29	12	2.26	0.04	0.44
	female	WT v COX.DKO	fixed	p Campilobacterota g Helicobacter	0.66	0.29	12	2.30	0.04	0.44
	female	WT v COX.DKO	fixed	p Firmicutes g Candidatus Stoquefichus	0.50	0.15	12	3.29	0.01	0.2
	female	WT v COX.DKO	fixed	p Patescibacteria g Candidatus Saccharimonas	0.74	0.21	12	3.45	0.00	0.2
	male	WT v COX.DKO	fixed	p Bacteroidota g Muribaculaceae	-0.08	0.03	10	-2.59	0.03	0.42
	male	WT v COX.DKO	fixed	p Firmicutes g Lachnospiraceae UCG-001	-0.94	0.34	10	-2.73	0.02	0.4
	male	WT v COX.DKO	fixed	p Proteobacteria g Parasutterella	-0.46	0.10	10	-4.46	0.00	0.11

Supplemental Figure 7

A

Separately Housed

Taxa	sex	term	estimate	std.error	df	statistic	p.value	fdr
p Bacteroidota f Prevotellaceae	Female	WT v COX.DKO	0.37	0.11	14	3.42	0.00	**0.057**
p Bacteroidota f Prevotellaceae	Male	WT v COX.DKO	0.24	0.08	14	3.14	0.01	**0.072**
p Bacteroidota g Muribaculum	Female	WT v COX.DKO	-0.24	0.13	14	-1.88	0.08	0.28
p Bacteroidota g Muribaculum	Male	WT v COX.DKO	-0.22	0.09	14	-2.41	0.03	0.18
p Bacteroidota o Bacteroidales	Female	WT v COX.DKO	0.44	0.25	14	1.78	0.10	0.29
p Bacteroidota o Bacteroidales	Male	WT v COX.DKO	0.48	0.23	14	2.14	0.05	0.22
p Bacteroidota s Bacteroides acidifaciens	Female	WT v COX.DKO	1.01	0.28	14	3.59	0.00	**0.055**
p Bacteroidota s Bacteroides sartorii	Female	WT v COX.DKO	0.47	0.20	14	2.34	0.03	0.18
p Bacteroidota s Bacteroides uniformis	Female	WT v COX.DKO	1.26	0.35	14	3.57	0.00	**0.055**
p Bacteroidota s uncultured organism	Male	WT v COX.DKO	0.28	0.09	14	3.10	0.01	**0.072**
p Bacteroidota s unidentified	Female	WT v COX.DKO	0.46	0.21	14	2.21	0.04	0.2
p Bacteroidota s unidentified	Male	WT v COX.DKO	0.32	0.12	14	2.68	0.02	0.12
p Firmicutes g Faecalibaculum	Male	WT v COX.DKO	-2.28	0.43	14	-5.30	0.00	**0.0092**
p Firmicutes g uncultured	Male	WT v COX.DKO	0.46	0.25	14	1.83	0.09	0.29
p Firmicutes s Lachnospiraceae	Female	WT v COX.DKO	1.26	0.55	14	2.30	0.04	0.18
p Firmicutes s Lachnospiraceae	Male	WT v COX.DKO	1.20	0.62	14	1.92	0.08	0.27
p Firmicutes s Lactobacillus intestinalis	Female	WT v COX.DKO	0.88	0.44	14	1.99	0.07	0.26
p Firmicutes s Lactobacillus intestinalis	Male	WT v COX.DKO	-2.46	0.57	14	-4.35	0.00	**0.027**
p Firmicutes s Lactobacillus johnsonii	Female	WT v COX.DKO	0.49	0.25	14	2.00	0.07	0.26
p Firmicutes s Lactobacillus murinus	Female	WT v COX.DKO	0.96	0.32	14	3.02	0.01	**0.075**
p Firmicutes s Lactobacillus murinus	Male	WT v COX.DKO	1.29	0.41	14	3.16	0.01	**0.072**
p Firmicutes s Lactobacillus reuteri	Female	WT v COX.DKO	0.42	0.18	14	2.32	0.04	0.18
p Firmicutes s Romboutsia ilealis	Female	WT v COX.DKO	-0.97	0.50	14	-1.96	0.07	0.26
p Firmicutes s Turicibacter sp.	Female	WT v COX.DKO	-2.89	0.82	14	-3.52	0.00	**0.055**
p Firmicutes s Turicibacter sp.	Male	WT v COX.DKO	-2.13	0.91	14	-2.35	0.03	0.18
p Firmicutes s uncultured bacterium	Female	WT v COX.DKO	-0.29	0.17	14	-1.77	0.10	0.29
p Firmicutes s uncultured bacterium	Male	WT v COX.DKO	-0.54	0.18	14	-2.95	0.01	**0.079**

B

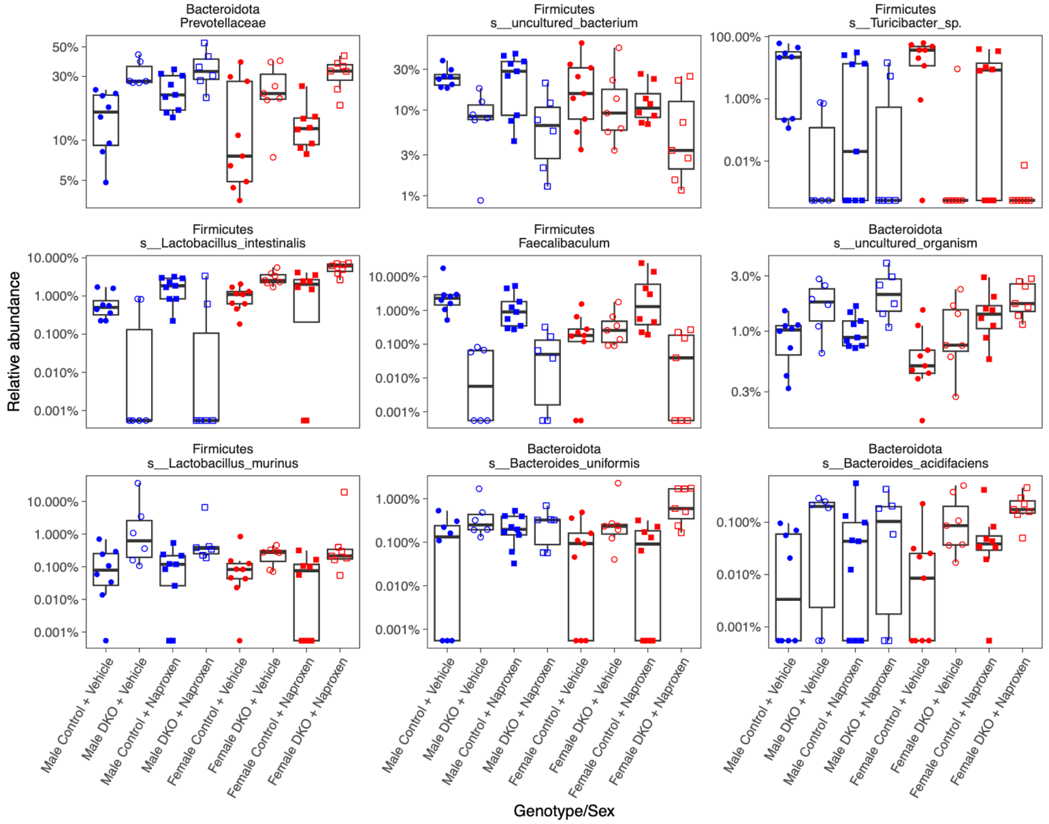

C

Co-Housed

Taxa	sex	term	estimate	std.error	df	statistic	p.value	fdr
p Bacteroidota f Prevotellaceae	Female	WT v COX.DKO	0.29	0.13	12	2.26	0.04	0.46
p Bacteroidota s Bacteroides sartorii	Female	WT v COX.DKO	0.78	0.32	12	2.47	0.03	0.46
	Male	WT v COX.DKO	0.53	0.27	10	1.95	0.08	0.46
p Bacteroidota s Bacteroides uniformis	Female	WT v COX.DKO	0.47	0.15	12	3.25	0.01	0.31
p Bacteroidota s mouse gut	Female	WT v COX.DKO	-0.24	0.09	12	-2.67	0.02	0.46
p Bacteroidota s uncultured organism	Female	WT v COX.DKO	0.18	0.08	12	2.15	0.05	0.46
p Campilobacterota s Helicobacter apodemus	Female	WT v COX.DKO	0.63	0.28	12	2.20	0.05	0.46
p Firmicutes f Ruminococcaceae	Female	WT v COX.DKO	-0.46	0.23	12	-1.96	0.07	0.46
p Firmicutes g Clostridia UCG-014	Female	WT v COX.DKO	0.37	0.20	12	1.81	0.10	0.46
p Firmicutes g Faecalibaculum	Male	WT v COX.DKO	-0.76	0.37	10	-2.06	0.07	0.46
p Firmicutes g Lachnospiraceae NK4A136 group	Male	WT v COX.DKO	-0.71	0.38	10	-1.87	0.09	0.46
p Firmicutes g Lactobacillus	Male	WT v COX.DKO	0.48	0.25	10	1.90	0.09	0.46
p Firmicutes s Lactobacillus murinus	Male	WT v COX.DKO	0.42	0.19	10	2.25	0.05	0.46
p Patescibacteria s uncultured bacterium	Female	WT v COX.DKO	0.74	0.21	12	3.48	0.00	0.31
p Proteobacteria s uncultured bacterium	Male	WT v COX.DKO	-0.52	0.23	10	-2.27	0.05	0.46

Supplemental Figure 8

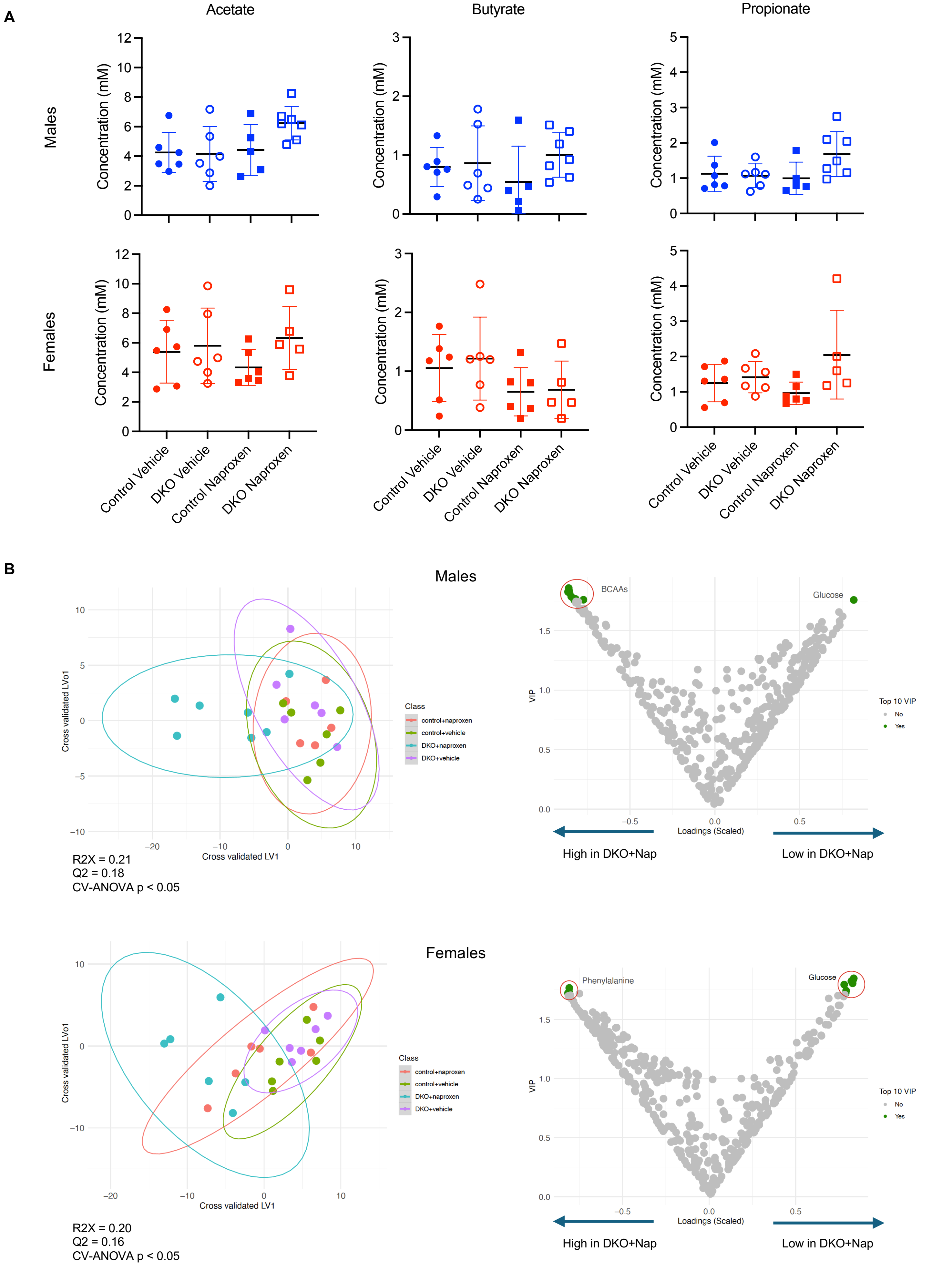

Supplemental Figure 9

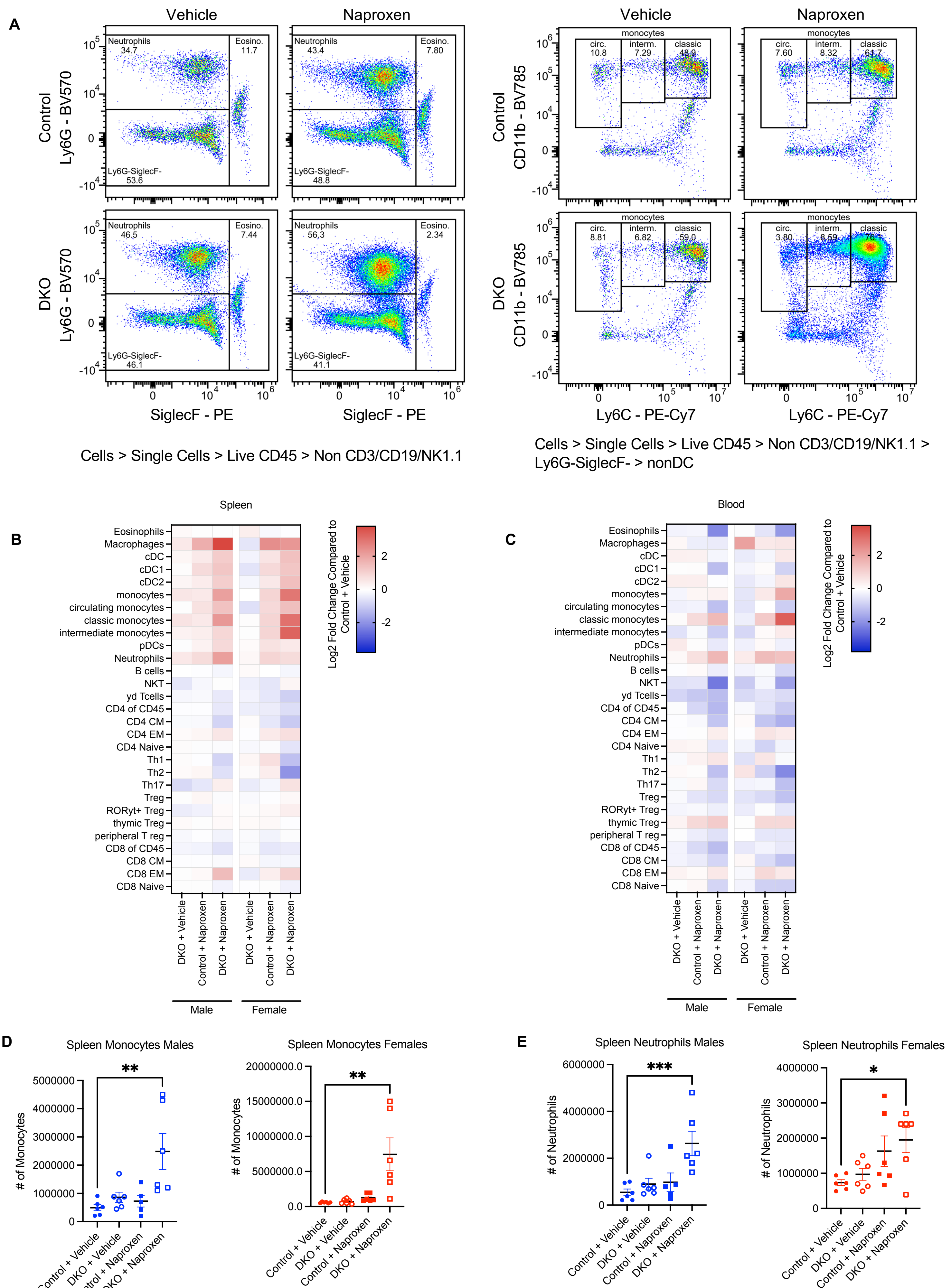

Supplemental Figure 10

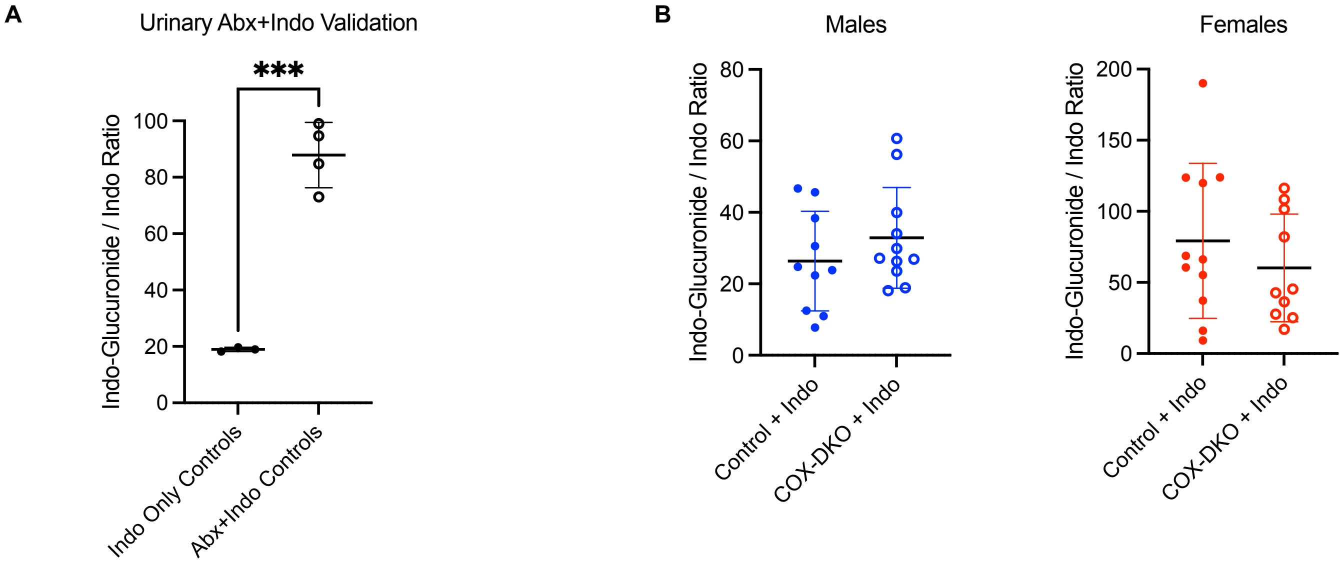

Supplemental Figure 11

A

Females Naproxen Day 0 vs Day 21
Differential Abundance OTU Level

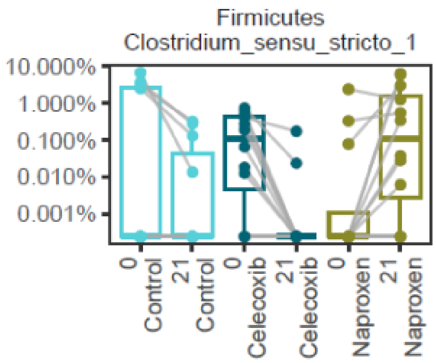

FDR 0.085

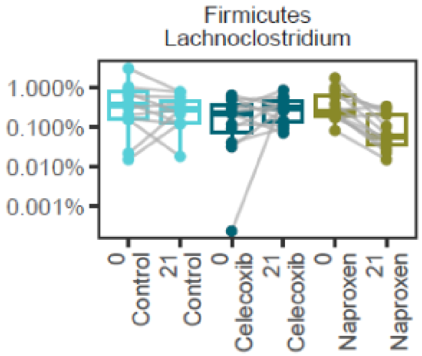

FDR 0.0097

B

Females Naproxen Day 0 vs Day 21
Differential Abundance ASV Level

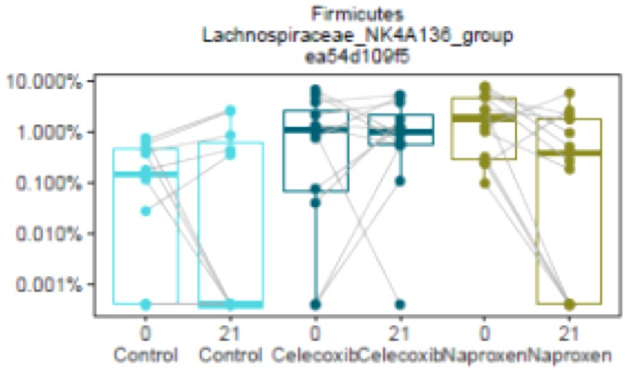

FDR 0.055

Supplemental Methods

Analysis from Human Gut Cell Atlas

The H5AD file containing November 19, 2024 version of Extended+ Pan-GI Cell Atlas(1) was downloaded from the Gut Cell Survey atlas (www.gutcellatlas.org), a part of Human Cell Atlas(2). The data were filtered to include only epithelial cells of small intestine belonging to donors aged between 18 and 74 years. This yielded 193,038 cells in total. Sex distribution was slightly biased towards males, with 45% of cells coming from female donors. Majority of filtered cells (147,244/193,038 = 76.2%) were collected as a part of a previous study(3). We investigated the expression of PTGS1 and PTGS2 across various intestinal epithelial cell subtypes and across four conditions: non-pathological, neighboring inflamed, Crohn's disease, and neighboring cancer. Plot only showing percentages of cells expressing PTGS1 or PTGS2. Microfold, INFLARE, and mucous gland neck cells were excluded due to low counts.

Real-Time Polymerase Chain Reaction Analysis of Gene Expression

Total RNA was isolated from lung and small intestine tissue samples using the Qiagen RNeasy Kit (Qiagen, Germantown, MD). Reverse transcription was performed using the Applied Biosystems HighCapacity cDNA Reverse Transcription Kit (Applied Biosystems, Waltham, MA). Real-time polymerase chain reaction was performed using ABI TaqMan primers and reagents on an ABI Prism 7500 Thermocycler according to the manufacturer's instructions. All mRNA measurements were normalized to *Hprt* mRNA levels using the $2^{-\Delta\Delta CT}$ method. The following TaqMan primers were used:

Ptgs1/Cox-1: Mm00477214_m1 (Life Tech / Invitrogen / ABI, Carlsbad, CA)
Ptgs2/Cox-2: Mm00478374_m1 (Life Tech / Invitrogen / ABI, Carlsbad, CA)
Hprt: Mm01545399_m1 (Life Tech / Invitrogen / ABI, Carlsbad, CA)

Protein Extraction and Western Blot Analyses

Small intestine samples were derived from *Cox*-DKO mice and *Cre*$^{-/-}$ control littermates challenged with LPS (1 mg/kg body weight), as described in Study 2 Design. Protein extraction of jejunal intestinal

tissue was performed using RIPA buffer (ThermoFisher Scientific) supplemented with protease (cOmplete mini protease inhibitor cocktail, Roche) and phosphatase inhibitors (PhosSTOP phosphatase inhibitor cocktail, Roche). 20 mg of tissue was homogenized in a TissueLyser II (QIAGEN) at 25 hz for 4 min. For Western blot analyses, 30 µg protein was separated by SDS-PAGE under reducing conditions, transferred to membranes, and probed with the appropriate antibodies. The antibodies used were: COX1 (Caymen Chemicals Company; 160109 at 1:200), COX2 (Caymen Chemicals Company; 160106 at 1:200), and HPRT (Santa Cruz Biotechnology; sc-20975 at 1:500). Membranes were developed with SuperSignal™ West Pico PLUS chemiluminescent substrate (ThermoFisher Scientific) and imaged with the Amersham™ Imager 600 (GE Healthcare Life Sciences). Densitometric analysis was conducted using ImageJ2.

Nuclear Magnetic Resonance Spectroscopy to Measure Microbiome-Associated Metabolites

All NMR experiments were performed using a Bruker Avance III HD NMR Spectrometer fitted with a 3mm TXI probe (Bruker Biospin, Billerica, MA). 50-100 mg per fecal sample were homogenized in water (10 µL per mg feces) and spun down at 10,000 x g for 10 minutes at 4°C; supernatant was transferred to a new tube, and spin step was repeated. Then supernatant was passed through a Durapore-PVDF 0.22 µm centrifugal filter (Merck Millipore Ltd., Cork, IRL) spun at 12,000 x g for 4 minutes at 4°C. Samples (180 µL) were dissolved in 20 mL of buffer containing D2O (Cortecnet Corp. New York, NY) and 0.26 mM internal standard (4,4-dimethyl-4-silapentane-1-sulfonic acid/DSS, Cambridge Isotope Laboratory, Andover, MA). Briefly, the first transient of a NOESY experiment was used for acquiring 1-dimensional NMR data with water signal saturation by continuous irradiation during relaxation delay (1 s) and mixing time (0.1 s). Each spectrum was acquired using 1024 scans, 76 K data points, and 14 ppm spectral width. The FIDs were zero-filled to 128 K; 0.1 Hz of linear broadening was applied followed by Fourier transformation. Metabolite levels in the spectra were quantified using a targeted profiling technique via Chenomx profiler V8.0 (Edmonton, AB, Canada). Multivariate data analysis was performed using Simca-P 17.0 (Sartorius Stedim, Aubagne, France). Principal Component Analysis was used to check the quality of data, followed by supervised Orthogonal Partial Least Square – Discriminant Analysis (OPLS-DA). OPLS-DA model was judged using Q^2(cum) (cross validated R^2 generated by 7-fold cross validation technique) and CV-ANOVA p values (< 0.05 denotes significant model).

Tissue Preparation and Flow Cytometry

To generate single cell suspension of splenocytes, freshly harvested murine spleen was placed on a 70 μm strainer and gently macerated through the filter with a 3 mL syringe plunger. Strainer was flushed with a total of 6 mL RPMI 1640 + 10% FBS, then cells were spun down at 500 x g for 5 minutes at room temperature, and supernatant was then removed. Pellet was resuspended in 5 mL RBC lysis buffer and incubated for 10 minutes at room temperature. Reaction was stopped by adding 10 mL PBS, and cells were spun down again at 500 x g for 5 minutes at room temperature. The RBC lysis – PBS wash cycle was repeated one more time until the pellet was no longer red. Pellet was then resuspended in 1 mL FACS buffer (PBS + 0.1% BSA). Cells were separated at a dilution of ~$1X10^6$/sample. Blood was prepared in the same manner, sans maceration step.

In preparation for staining, cells were pre-incubated with TruStain FcX™ PLUS (anti-mouse CD16/32) Antibody (BioLegend) and Zombie UV Fixable Viability dye (BioLegend). Two separate staining panels were used. The first panel included antibodies for CD172a, CD317, Ly6C, SiglecF, CD11b, CD64, CD3, CD19, NK1.1, CD11c, Ly6G, XCR1, MHCII/IA-IE, CD45, F4/80, and B220, and the second panel included antibodies for CD19, TCRγδ, CD3, CD44, NK1.1, CD127, CD62L, CD8b, CD45, CD4, CD8a, GATA3, Helios, T-bet, RORγt, and FoxP3; see tables below for details. Samples were fixed with Foxp3/Transcription Factor Staining Buffer Set (eBioscience). For transcription factors staining, cells were permeabilized and stained in the Foxp3/Transcription Factor Staining Buffer Set at 4°C. Samples were recorded on a Cytek Aurora (Cytek Biosciences) and analyzed using FlowJo. Details for gating strategies and analyses have been described previously.(4)

Myeloid Panel			
**Fluorophore**	**Antibody**	**Catalog #**	**Dilution**
PerCP/Cy5	CD172a	BioLegend #144009	1:100
AF488	CD317	BioLegend #127012	1:200
PE-Cy7	Ly6C	BioLegend #128017	1:100
PE	SiglecF	BioLegend #155505	1:100
BUV785	CD11b	BioLegend #101243	1:100
BV711	CD64	BioLegend #139311	1:200
BV650	CD3 / CD19 / NK1.1	BioLegend #100229 / BioLegend #115541 / BioLegend #108736	1:50
BV605	CD11c	BioLegend #117334	1:100
BV570	Ly6G	BioLegend #127629	1:100
BV510	XCR1	BioLegend #148218	1:100
PB	MHCII/IA-IE	BioLegend #107620	1:200
UV Zombie	Live	BioLegend #423108	1:200
BUV496	CD8a	BD #750024	1:100
BUV395	CD45	BD #564279	1:200
APC-Cy7	F4/80	BioLegend #123117	1:100
AF700	B220	BioLegend #103232	1:200

T Cell Panel				
**Fluorophore**	**Antibody**	**Catalog #**	**TF**	**Dilution**
PerCP/Cy5	CD19	Invitrogen 45-0193-82		1:100
AF488	GATA3	BD #560163	X	1:100
PE-Cy7	Helios	BioLegend #137236	X	1:100
PE/CF594	TCRyd	BD #563532		1:200
PE	Tbet	BioLegend #644810	X	1:50
BUV785	CD3	BioLegend #100232		1:100
BV711	CD44	BioLegend #103057		1:200
BV650	NK1.1	BioLegend #108736		1:200
BV605	CD127	BioLegend #135041		1:100
BV570	CD62L	BioLegend #104433		1:100
BV510	CD8b	BioLegend #126631		1:200
BV421	RORyt	BD #562894	X	1:100
UV Zombie	Live	BioLegend #423108		1:200
BUV395	CD45	BD #564279		1:200
APC-Cy7	CD4	BioLegend #100414		1:200
AF700	CD8a	BioLegend #100730		1:200
APC	FoxP3	Invitrogen 17-5773-82	X	1:100

LC-MS/MS Analysis of Indomethacin and its Metabolite (Indomethacin Acyl- β-D-Glucuronide)

Urinary indomethacin and indomethacin acyl- β-D-glucuronide were measured as described previously.(5) Briefly, 40 µL d4-Indomethacin (1 ng/µL) was spiked into the samples and brought to 1 mL by adding 920 µL water. Just before loading the sample for solid phase extraction (SPE), 20 µL formic acid was added to the mixture. SPE was performed using Strata-X 33µm polymeric reversed phase cartridges (Phenomenex, 8B-S100-TAK) by employing following steps: 1 mL methanol was added, followed by 0.25 mL water. The sample was loaded, washed with 1 mL water, and eluted with 1 mL methanol. The sample was dried and reconstituted in 100 µL of 10% acetonitrile. Separation of the compounds was carried out using a Waters ACQUITY UPLC system with an ultra-performance liquid chromatography (UPLC) column, 2.1 × 150 mm with 1.7 µm particles (Waters ACQUITY UPLC CSH C18) coupled with Waters TQS triple quadrupole instrument operated in electrospray negative ion mode. The precursor to product ion mass transitions used for indomethacin and indomethacin acyl- β-D-glucuronide were *m/z* 356.1/312.1 and *m/z* 532.1/193.1 using d4-indomethancin as an internal standard (*m/z* 360.1/316.1). Ten-point calibration samples (100 ng/µL, 50 ng/µL, 25 ng/µL, 12.5 ng/µL, 6.25 ng/µL, 3.125 ng/µL, 1.56 ng/µL, 0.78 ng/µL, 0.39 ng/µL, 0.195 ng/µL) were used for measuring indomethacin and its metabolite. The ratio of indomethacin acyl-β-D-glucuronide to indomethacin was determined using standard curves prepared in pooled mouse urine that was free of indomethacin and its metabolite.

References

1. Oliver AJ, Huang N, Bartolome-Casado R, Li R, Koplev S, Nilsen HR, et al. Single-cell integration reveals metaplasia in inflammatory gut diseases. *Nature.* 2024;635(8039):699-707.
2. Rood JE, Wynne S, Robson L, Hupalowska A, Randell J, Teichmann SA, et al. The Human Cell Atlas from a cell census to a unified foundation model. *Nature.* 2025;637(8048):1065-71.
3. Kong L, Pokatayev V, Lefkovith A, Carter GT, Creasey EA, Krishna C, et al. The landscape of immune dysregulation in Crohn's disease revealed through single-cell transcriptomic profiling in the ileum and colon. *Immunity.* 2023;56(2):444-58.e5.
4. Dallari S, Heaney T, Rosas-Villegas A, Neil JA, Wong SY, Brown JJ, et al. Enteric viruses evoke broad host immune responses resembling those elicited by the bacterial microbiome. *Cell Host Microbe.* 2021;29(6):1014-29.e8.
5. Liang X, Bittinger K, Li X, Abernethy DR, Bushman FD, and FitzGerald GA. Bidirectional interactions between indomethacin and the murine intestinal microbiota. *Elife.* 2015;4:e08973.
