# Supplementary material for: Concomitant COX-1 and COX-2 suppression is not sufficient to induce enteropathy associated with chronic NSAID use
Source: J Clin Invest. 2026 Jan 27;136(6):e190575. doi: 10.1172/JCI190575 (PMC12987628; doi:10.1172/JCI190575)

Unedited blot: Intestine stimulated blot COX2

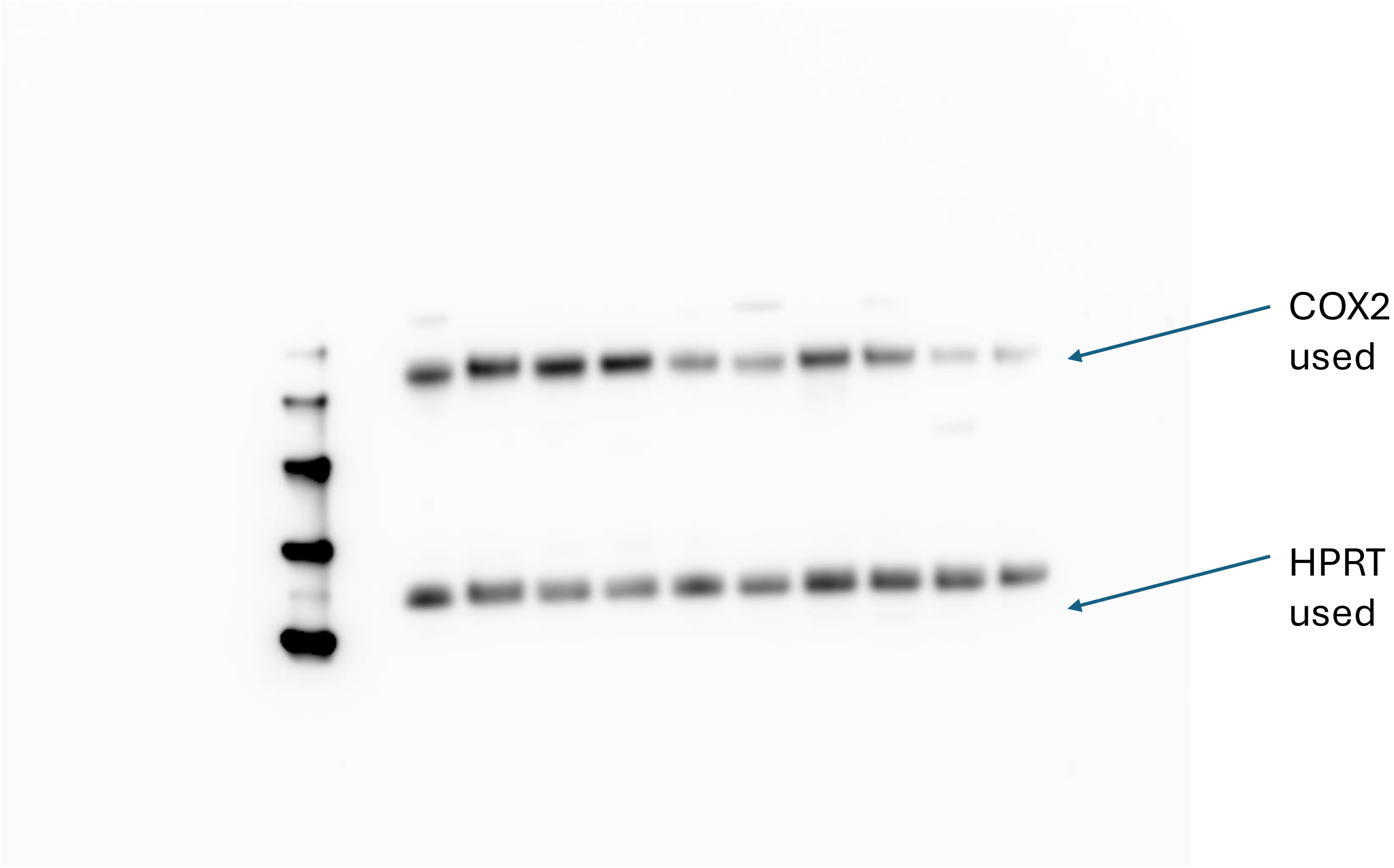

Unedited blot: Intestine stimulated blot COX1

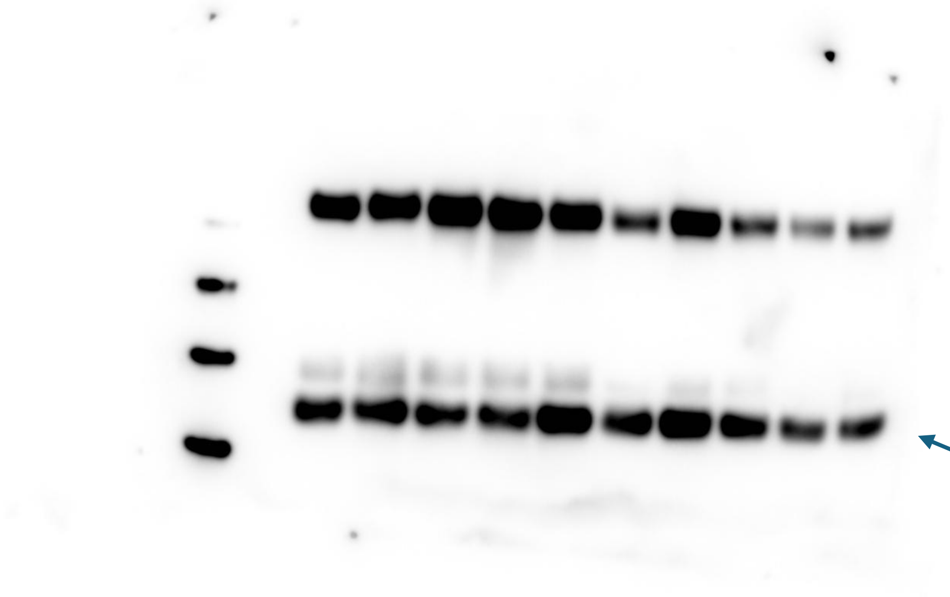

HRPT
used

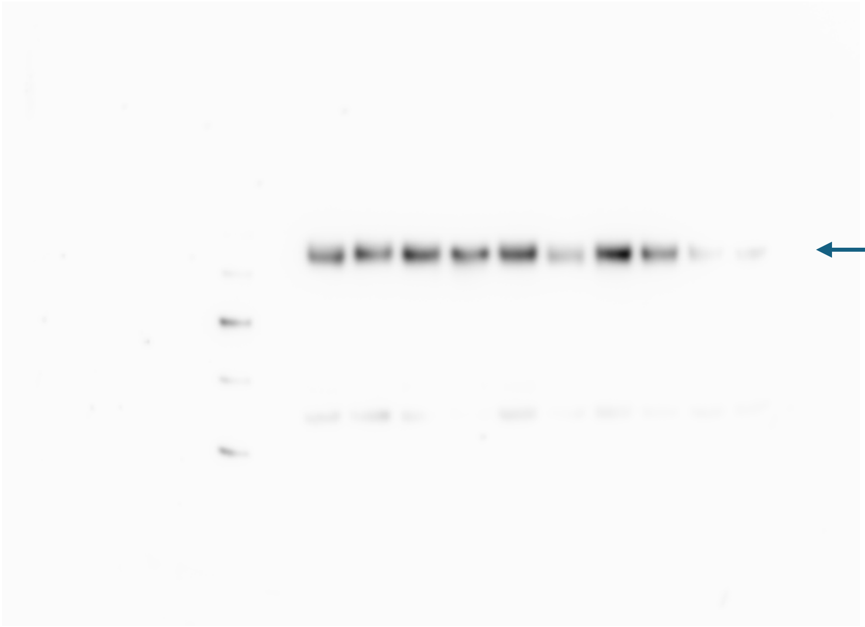

COX1
used

Unedited blot: Intestine unstimulated blot COX2

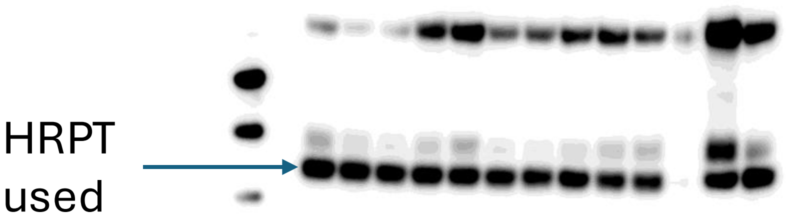

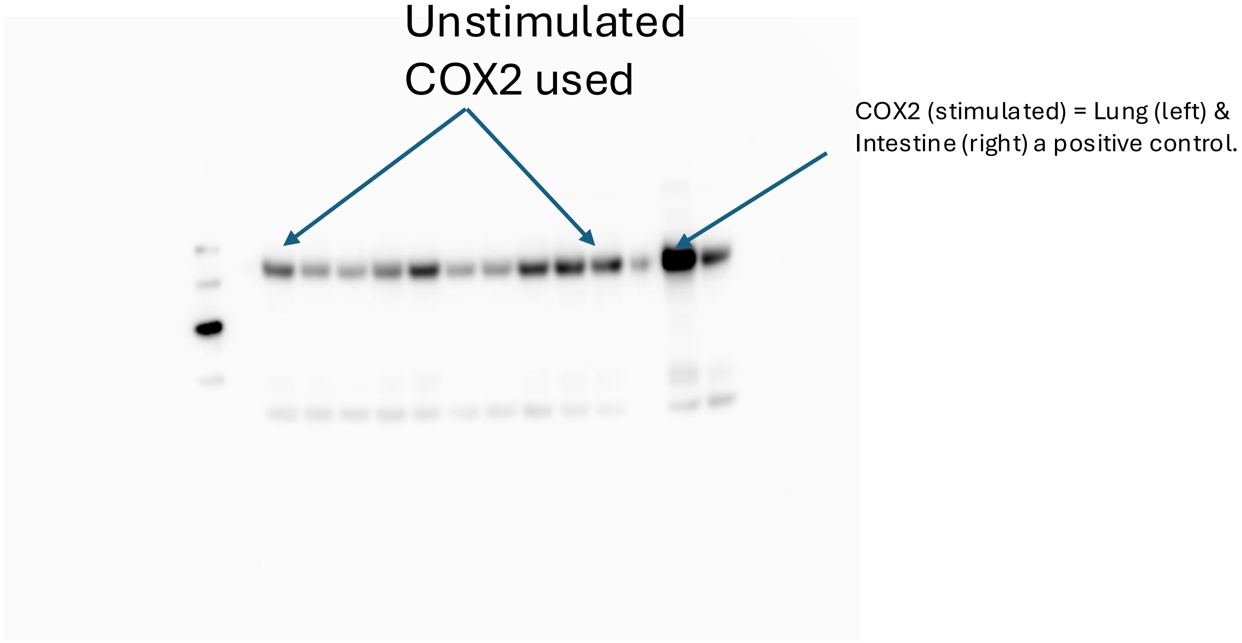

Unedited blot: Intestine unstimulated blot COX1

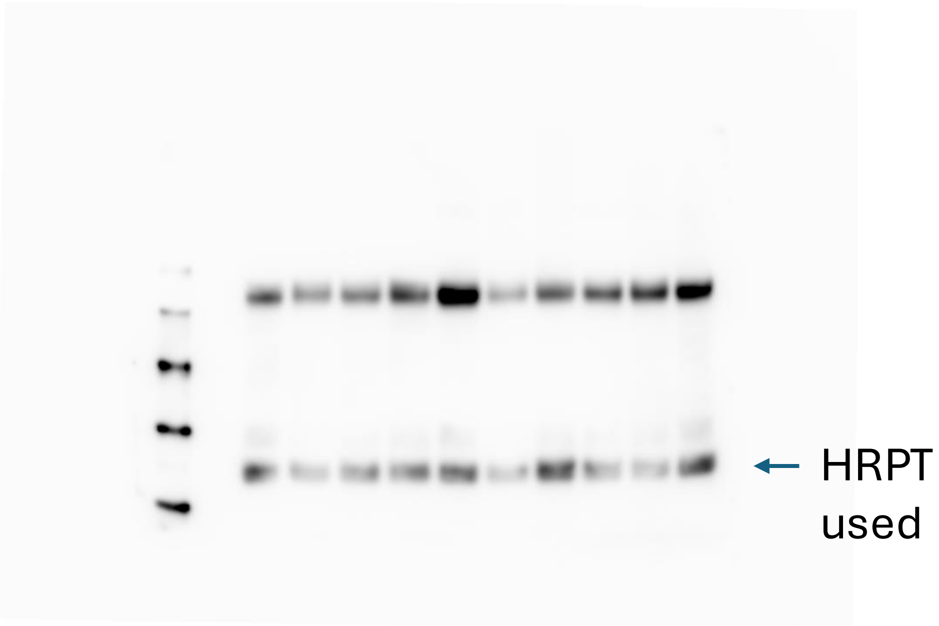

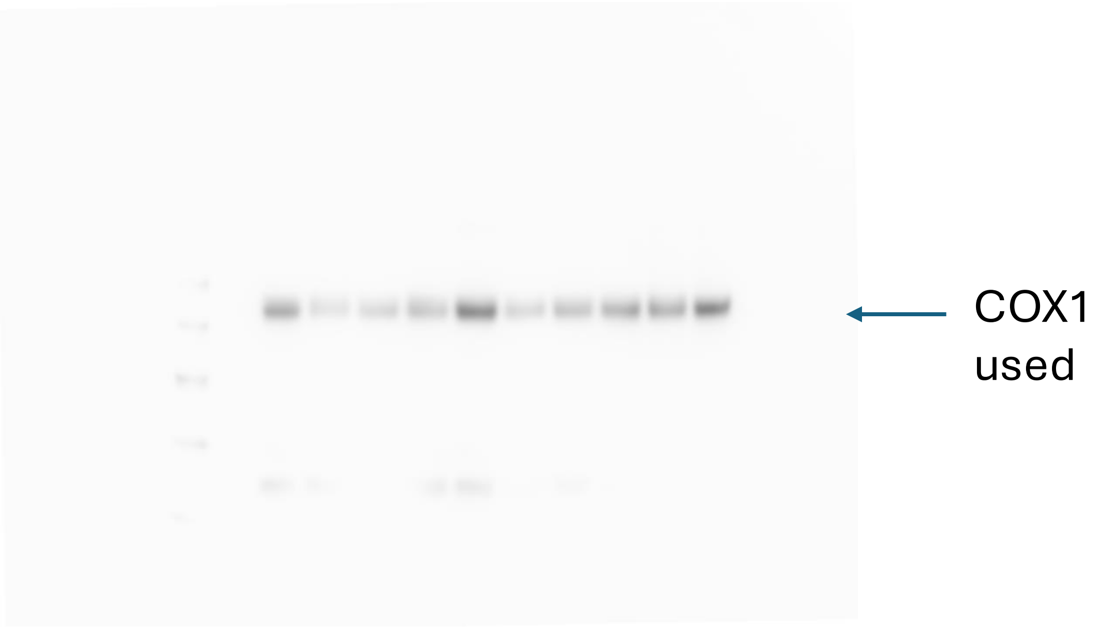

Supplement: Unedited blot and gel images [file jci-136-190575-s060.pdf]
